# Insights into the molecular phylogeny and morphology of three novel *Dothiora* species, along with a worldwide checklist of *Dothiora*

**DOI:** 10.3389/fcimb.2024.1367673

**Published:** 2024-04-19

**Authors:** Chanokned Senwanna, Sinang Hongsanan, Surapong Khuna, Jaturong Kumla, Manzura Yarasheva, Yusufjon Gafforov, Aziz Abdurazakov, Nakarin Suwannarach

**Affiliations:** ^1^Office of Research Administration, Chiang Mai University, Chiang Mai, Thailand; ^2^Center of Excellence in Microbial Diversity and Sustainable Utilization, Chiang Mai University, Chiang Mai, Thailand; ^3^Guangdong Provincial Key Laboratory for Plant Epigenetics, Shenzhen Key Laboratory of Microbial Genetic Engineering, College of Life Science and Oceanography, Shenzhen University, Shenzhen, China; ^4^Department of Education and Training Management, Tashkent International University of Education, Tashkent, Uzbekistan; ^5^Central Asian Center for Development Studies, New Uzbekistan University, Tashkent, Uzbekistan; ^6^Mycology Laboratory, Institute of Botany, Academy of Sciences of Republic of Uzbekistan, Tashkent, Uzbekistan; ^7^Department of Ecology and Botany, Faculty of Natural Sciences, Andijan State University, Andijan, Uzbekistan

**Keywords:** Asia, Dothideomycetes, Dothideales, fungal taxonomy, new species, saprobic fungi

## Abstract

Most species of *Dothiora* are known from the dead parts of various host plants as saprobic fungi in terrestrial habitats occurring in tropical and temperate regions. In the present study, samples of *Dothiora* were collected from dead twigs and branches of *Capparis spinosa*, *Rhaponticum repens*, and an unknown angiosperm plant from the Tashkent and Jizzakh regions of Uzbekistan. Multi-gene phylogenetic analyses based on a combined ITS, LSU, SSU, *TEF1*, and *TUB2* sequence data revealed their taxonomic positions within the Dothideaceae. Three new species of *Dothiora*, namely, *Dothiora capparis*, *Dothiora rhapontici*, and *Dothiora uzbekistanica* were proposed by molecular and morphological data. Likewise, the phylogenetic relationship and morphology of *Dothiora* are discussed. In addition, we provide a list of accepted *Dothiora* species, including host information, distribution, morphology descriptions, and availability of sequence data, to enhance the current knowledge of the diversity within *Dothiora*.

## Introduction

Dothideales Lindau is an order in the class Dothideomycetes that comprises four families, including Dothideaceae, Neocelosporiaceae, Saccotheciaceae, and Zalariaceae (Hongsanan et al., 2020; Wijayawardene et al., 2022). Members of this order are saprobic and occasionally pathogenic to plants in terrestrial habitats and humans, including house dust (Thambugala et al., 2014; Crous et al., 2018a; Hongsanan et al., 2020). In addition, some species have been used as potential biocontrol for pest management (Zajc et al., 2019; Cignola et al., 2023). To date, 26 genera were accepted in Dothideales, while six genera were proposed in Dothideales genera *incertae sedis* (Wijayawardene et al., 2022). Dothideaceae Chevall. was introduced to accommodate the genus *Dothidea*, with *D. gibberulosa* as the type species (Chevallier, 1826; Fuckel, 1869). Currently, 14 genera are accepted in Dothideaceae, *viz*., *Delphinella* (Sacc.) Kuntze, *Dictyodothis* Theiss. & Syd., *Dothidea* Fr., *Dothiora* Fr., *Endoconidioma* Tsuneda, *Endodothiora* Petr., *Kabatina* R. Schneid. & Arx, *Neocylindroseptoria* Thambug. & K.D. Hyde, *Neodothiora* Crous, G.C. Adams & Winton, *Phaeocryptopus* Naumov, *Plowrightia* Sacc., *Stylodothis* Arx & E. Müll., *Sydowia* Bres., and *Uleodothis* Theiss. & Syd (Wijayawardene et al., 2022).

*Dothiora* was established by Fries (1894), typified by *D. pyrenophora* Berk. ex Sacc. The sexual morph of *Dothiora* is characterized by immersed to erumpent ascostromata, lacking pseudoparaphyses, eight or more spored, bitunicate asci, hyaline to yellow or pale brown, and one septate or muriform ascospores (Thambugala et al., 2014; Crous and Groenewald, 2017). *Dothiora* has dothichiza-like asexual morph via culture studies, which is characterized by pycnidial conidiomata, phialidic conidiogenous cells, hyaline, aseptate conidia, forming a hormonema-like synasexual morph (Thambugala et al., 2014; Crous and Groenewald, 2016; Crous and Groenewald, 2017). Earlier, *Dothiora* was treated in different families, such as Dothideaceae and Dothioraceae, by several authors (Barr, 1972; Barr, 1979; Barr, 1987; Froidevaux, 1972; Luttrell, 1973; von Arx and Müller, 1975; Hawksworth et al., 1995; Lumbsch and Huhndorf, 2010); however, Dothioraceae was synonymized under Dothideaceae based on its phylogenetic placement within Dothideales (Thambugala et al., 2014). Although 89 epithets are listed in Index Fungorum (accessed on 13 March 2024), most species have not been well studied since their introduction, and many species have been transferred to other genera such as *Dothiorella*, *Myriangium*, *Protoscypha*, and *Saccothecium*. Furthermore, only 28 *Dothiora* species have been sequenced and confirmed their phylogenetic placements (Hyde et al., 2020; Boonmee et al., 2021). *Dothiora* species are distributed worldwide on woody plants in terrestrial habitats as saprobes and pathogens causing leaf spots or possibly weak pathogens on stressed plant tissues (Crous and Groenewald, 2016; Crous and Groenewald, 2017; Hyde et al., 2020). Not only *Dothiora infuscans* Rodr.-Andr., Stchigel, Guarro & Cano is reported on the blackened wall of an industrial warehouse (Crous et al., 2018a), but also *Dothiora* sp. is recorded as an endophytic fungus that produces compounds with cytotoxic activity against cancer cell lines (Pérez-Bonilla et al., 2017).

Most of the *Dothiora* species are reported from Europe and North America (Hyde et al., 2020). Targeting underexplored regions such as Central Asia, including Uzbekistan, might be helpful for the discovery of new fungi (Gafforov, 2017; Cheek et al., 2020). Recent studies have led to the discovery of several new genera and species of ascomycetous microfungi in Uzbekistan (Gafforov and Rakhimov, 2017; Pem et al., 2018; Pem et al., 2019a; Pem et al., 2019b; Gafforov et al., 2019; Abdurazakov et al., 2021; Appadoo et al., 2021; Htet et al., 2021; Lestari et al., 2021; Aluthmuhandiram et al., 2022; Dong et al., 2023). However, *Dothiora* is still poorly known in Asia including Central Asian regions. The aim of the present study was to clarify the taxonomic position of *Dothiora* and to identify new taxa through multi-gene phylogeny and morphological examination. Fresh specimes collected from Uzbekistan were examined and their DNA sequence data were obtained for use in multi-gene phylogenetic analyses. Moreover, an updated list of *Dothiora* species worldwide is also provided.

## Materials and methods

### Sample collection and specimen examination

Specimens were collected from *Capparis spinosa*, *Rhaponticum repens*, and an unknown angiosperm plant from the Tashkent and Jizzakh regions of Uzbekistan. The collected specimens were brought to the laboratory in small plastic bags. Ascomata were sectioned by hand, examined, and captured under a Nikon SMZ800N stereomicroscope. The slides were prepared by mounting the materials in double-distilled water (ddH_2_O), lactophenol, and Indian ink stain. The micro-morphological characters were examined and captured using a Nikon DS-Ri2 camera connected with a Nikon ECLIPSE Ni (Tokyo, Japan) compound microscope. The measurement of structures was done by the Tarosoft^®^ Image Framework program (v.0.9.0.7). Adobe Photoshop Version: 22.4.2 (Adobe Systems U.S.A.) was used to make the photographic plates. The specimens were deposited in the Herbarium of the Department of Biology (CMUB), Faculty of Science, Chiang Mai University, Thailand, and the Tashkent Mycological Herbarium (TASM) of the Institute of Botany, Uzbekistan Academy of Sciences, Uzbekistan.

### DNA extraction, PCR amplification, and sequencing

To obtain pure cultures, single ascospore isolation was carried out following the methods of Senwanna et al. (2019) and Senanayake et al. (2020). However, no germinated ascospores were found on Petri dishes containing 2% malt extract agar (MEA; Gibco, Life Technologies Corporation, USA), 2% water agar (WA), and potato dextrose agar (PDA; BD Difco™, Becton, Dickinson and Company, USA) after incubation at 25°C to 30°C in the dark for 24–96 h. Fungal fruiting bodies, thus, were picked up and placed in a 1.5-mL sterilized tube. Genomic DNA was directly extracted, using E.Z.N.A.^®^ Genomic DNA Isolation Kits (OMEGA Bio-Tek, Georgia) following the manufacturer’s protocol. Polymerase chain reaction (PCR) amplification was carried out using the primer pairs as follows: ITS5 and ITS4 (White et al., 1990) to amplify the partial gene regions of internal transcribed spacers (ITS); LR0R and LR5 (Vilgalys and Hester, 1990) to amplify the 28S large subunit (LSU); NS1 and NS4 (White et al., 1990) to amplify the 18S small subunit (SSU); and EF1-728F (Carbone and Kohn, 1999) and EF2 (O’Donnell et al., 1998) to amplify the protein coding region for the translation elongation factor 1-alpha gene (*TEF1*). The PCR mixture contained 6 µL of double-distilled water (ddH_2_O),10 μL of 2 × Quick TaqTM HS DyeMix (TOYOBO, Japan), 2 µL of genomic DNA, and 1 μL of each forward and reverse primer. The PCR thermal cycle programs for ITS, LSU, and SSU amplification were as follows: initial denaturing step of 95°C for 5 min, followed by 35 cycles of denaturation at 94°C for 30 s, annealing at 52°C for 45 s, elongation at 72°C for 1 min, and final extension at 72°C for 10 min. The PCR thermal cycle programs for *TEF1* amplification were as follows: initial denaturing step of 94°C for 5 min, followed by 40 cycles of denaturation at 94°C for 30 s, annealing at 56°C for 30 s, elongation at 72°C for 1 min, and final extension at 72°C for 10 min. PCR products were examined on 1% agarose electrophoresis gels under UV light. PCR products were purified using the PCR clean-up Gel extraction NucleoSpin^®^ Gel and PCR Clean-up Kit (Macherey-Nagel, Germany) following the manufacturer’s protocol. PCR fragments were performed and sequenced at 1st BASE Company (Kembangan, Malaysia).

### Phylogenetic analyses

The generated sequence data were assembled using SeqMan 5.00, and the consensus sequences were subjected to BLASTn searches of the NCBI nucleotide database (http://blast.ncbi.nlm.nih.gov/; accessed on 2 November 2023) to determine their most probable closely related taxa. The representative taxa used in the analyses were selected from GenBank based on the BLASTn searches and recently published data (Boonmee et al., 2021; Gao et al., 2021) (**Table 1**). Each gene alignment was carried out with MAFFT version 7 (Katoh et al., 2019; http://mafft.cbrc.jp/alignment/server/; accessed on 6 November 2023) and was improved manually where necessary. The phylogenetic tree was carried out using the maximum likelihood (ML). The single gene datasets were then combined using BioEdit v.7.0.9.1 (Hall, 1999). The final alignments of the combined ITS, LSU, SSU, *TEF1*, and beta-tubulin (*TUB2*) datasets were analyzed, and the phylogenetic trees were inferred based on ML and Bayesian inference (BI) analyses.

**Table 1:** Taxa names, strain numbers, and GenBank accession numbers of sequences used in the phylogenetic analyses of this study.

Fungal Taxa	Strain	GenBank Accession Numbers					References
		ITS	LSU	SSU	*TEF1*	*TUB2*	
*Coniozyma leucospermi*	CBS 114035	AY720707	N/A	AY720711	N/A	N/A	Lennox et al. (2004)
*Coniozyma leucospermi*	CBS 111289	EU552113	EU552113	N/A	N/A	N/A	Marincowitz et al. (2008)
*Delphinella balsameae*	DJO-B-080615-A4	KY997059	N/A	N/A	KY997060	MF034404	Guertin et al. (2019)
*Delphinella strobiligena*	CBS 735.71	MH860318	MH872074	DQ471029	N/A	N/A	Spatafora et al. (2006); Vu et al. (2019)
*Dothidea berberidis*	CBS 186.58	EU167601	EU167601	EU167601	N/A	N/A	Simon et al. (2009)
*Dothidea hippophaeos*	AFTOL-ID 919	N/A	DQ678048	N/A	DQ677887	N/A	Schoch et al. (2006b)
*Dothidea insculpta*	CBS 189.58	AF027764	DQ247802	DQ247810	DQ471081	N/A	Jacobs and Rehner (1998); Schoch et al. (2006a)
*Dothidea mueller*	CBS 191.58	EU167593	EU167593	EU167593	N/A	N/A	Simon et al. (2009)
*Dothidea ribesia*	CPC 30638	KY929140	KY929173	N/A	KY929192	KY929205	Crous and Groenewald (2017)
*Dothidea ribesia*	CPC 30689	KY929141	KY929174	N/A	KY929193	KY929206	Crous and Groenewald (2017)
*Dothidea sambuci*	AFTOL-ID 274^T^	AY883094	AY544681	AY544722	DQ497606	N/A	Lutzoni et al. (2004); Shoemaker and Hambleton (2005)
*Dothiora agapanthi*	CPC 20600T	KU728498	KU728537	N/A	KU728578	KU728617	Crous and Groenewald (2016)
*Dothiora aloidendri*	CPC 38535	MW175347	MW175387	N/A	MW173123	MW173138	Crous et al. (2020)
*Dothiora buxi*	MFLU 15-3404^T^	KX765294	KX765295	N/A	N/A	N/A	Hyde et al. (2016)
*Dothiora bupleuricola*	CBS 112.75^T^	KU728499	KU728538	N/A	KU728579	KU728618	Crous and Groenewald (2016)
*Dothiora cactacearum*	CBS 142492^T^	KY929143	KY929176	N/A	KY929195	KY929208	Crous and Groenewald (2017)
*Dothiora cactacearum*	CPC 15587	KY929144	KY929177	N/A	KY929196	KY929209	Crous and Groenewald (2017)
*Dothiora cannabinae*	AFTOL-ID 1359^T^	AJ244243	DQ470984	DQ479933	DQ471107	N/A	Spatafora et al. (2006); De Hoog et al. (1999)
*Dothiora capparis*	TASM 6169^T^	PP086677	PP086685	PP086692	PP084937	N/A	This study
*Dothiora capparis*	CMUB40036	PP086678	PP086686	N/A	PP084938	N/A	This study
*Dothiora capparis*	CMUB40037	PP086679	PP086687	PP086693	PP093832	N/A	This study
*Dothiora capparis*	CMUB40038	PP086680	PP086688	PP086694	PP093833	N/A	This study
*Dothiora ceratoniae*	CBS 477.69^T^	KF251151	KF251655	N/A	KF253111	KF252649	Quaedvlieg et al. (2013)
*Dothiora coronillae*	MFLU 17-0005	MF443252	N/A	N/A	N/A	N/A	Hyde et al. (2017)
*Dothiora coronillicola*	MFLUCC 17-1007	MZ571207	MZ571206	N/A	N/A	N/A	Boonmee et al. (2021)
*Dothiora cytisi*	MFLUCC 14-0970^T^	KU248848	KU248849	KU248850	N/A	N/A	Li et al. (2016)
*Dothiora elliptica*	CBS 736.71^T^	KU728502	KU728541	N/A	GU349013	N/A	Schoch et al. (2009); Crous and Groenewald (2016)
*Dothiora europaea*	CBS 740.71	MH860322	MH872078	N/A	N/A	N/A	Vu et al. (2019)
*Dothiora infuscans*	FMR 16326 ^T^	LT993342	LT993345	N/A	N/A	N/A	Crous et al. (2018a)
*Dothiora laureolae*	CBS 744.71^T^	KU728503	KU728542	N/A	N/A	N/A	Crous and Groenewald (2016)
*Dothiora mahoniae*	CBS 264.92^T^	MH862357	MH874022	N/A	N/A	N/A	Vu et al. (2019)
*Dothiora maculans*	CBS 299.76	KU728504	KU728543	N/A	KU728582	KU728621	Crous and Groenewald (2016)
*Dothiora maculans*	CBS 301.76	KU728505	KU728544	N/A	KU728583	KU728622	Crous and Groenewald (2016)
*Dothiora maculans*	CBS 302.76	KU728506	KU728545	N/A	KU728584	KU728623	Crous and Groenewald (2016)
*Dothiora oleae*	CBS 152.71	KU728508	KU728547	N/A	KU728586	KU728625	Crous and Groenewald (2016)
*Dothiora oleae*	CBS 235.57	KU728509	KU728548	N/A	KU728587	KU728626	Crous and Groenewald (2016)
*Dothiora oleae*	CBS 472.69	KU728510	KU728549	N/A	KU728588	KU728627	Crous and Groenewald (2016)
*Dothiora omaniana*	SQUCC 13293	MT077213	MT077209	N/A	MT081204	MT081205	Hyde et al. (2020)
*Dothiora phaeosperma*	CBS 870.71	KU728512	KU728550	N/A	N/A	N/A	Crous and Groenewald (2016)
*Dothiora phillyreae*	CBS 473.69^T^	KU728513	EU754146	N/A	KU728590	KU728629	De Gruyter et al. (2009); Crous and Groenewald (2016)
*Dothiora prunorum*	CBS 933.72^T^	AJ244248	KU728551	N/A	N/A	N/A	De Hoog et al. (1999); Crous and Groenewald (2016)
*Dothiora pyrenophora*	CPC 30632^T^	KY929145	KY929178	KY929125	KY929203	KY929210	Crous and Groenewald (2017)
*Dothiora pyrenophora*	CPC 30634	N/A	KY929179	N/A	KY929204	KY929211	Crous and Groenewald (2017)
*Dothiora rhamni-alpinae*	CBS 745.71	MH860327	MH872082	N/A	N/A	N/A	Vu et al. (2019)
*Dothiora rhapontici*	TASM 6170^T^	PP086681	PP086689	PP086695	PP084939	N/A	This study
*Dothiora rhapontici*	CMUB40040	PP086682	PP086690	PP086696	PP084940	N/A	This study
*Dothiora rhapontici*	CMUB40041	PP086683	N/A	N/A	PP084941	N/A	This study
*Dothiora schizospora*	CBS:189.55	MH857439	MH868980	N/A	N/A	N/A	Vu et al. (2019)
*Dothiora sorbi*	CBS 742.71	KU728514	KU728552	N/A	N/A	N/A	Crous and Groenewald (2016)
*Dothiora spartii*	MFLU 15-3469	MF443250	MF443253	N/A	N/A	N/A	Hyde et al. (2017)
*Dothiora uzbekistanica*	TASM 6171^T^	PP086684	PP086691	PP086697	PP084936	N/A	This study
*Dothiora viburnicola*	CBS 274.72^T^	KU728515	KU728554	N/A	KU728591	N/A	Crous and Groenewald (2016)
*Dothiora viticola*	CBS 140676	NR_137620	MH878164	N/A	N/A	N/A	Vu et al. (2019)
*Endoconidioma rosae-hissaricae*	MFLUCC 17-0821^T^	MG828898	MG829008	MG829119	N/A	N/A	Wanasinghe et al. (2018)
*Endoconidioma populi*	UAMH 10297	AY604526	EU981287	AY604526	N/A	N/A	Tsuneda et al. (2004)
*Endoconidioma populi*	UAMH 10902	HM185487	HM185488	N/A	N/A	N/A	Tsuneda et al. (2010)
*Hormonema carpetanum*	TRN31	AY616206	N/A	N/A	N/A	N/A	Bills et al. (2004)
*Hormonema dematioides*	C1-07/07/98	AJ278927	N/A	N/A	N/A	N/A	Tsuneda et al. (2010)
*Hormonema macrosporum*	CBS 536.94^T^	AJ244247	MH874128	N/A	N/A	N/A	De Hoog et al. (1999); Vu et al. (2019)
*Kabatina juniperi*	CBS 466.66	AY616212	N/A	N/A	N/A	N/A	Bills et al. (2004)
*Kabatina thujae*	CBS 238.66	MH858786	MH870424	N/A	N/A	N/A	Vu et al. (2019)
*Neocylindroseptoria corymbiae*	CBS 145060^T^	MK047431	MK047482	N/A	N/A	N/A	Crous et al. (2018b)
*Neocylindroseptoria pistaciae*	CBS 471.69^T^	KF251152	KF251656	N/A	KF253112	KF252650	Quaedvlieg et al. (2013)
*Neodothiora populina*	CPC 39399	MW175365	MW175405	N/A	MW173127	MW173142	Crous et al. (2020)
*Phaeocryptopus nudus*	CBS 268.37	EU700371	GU301856	GU296182	GU349034	EU747283	Park et al. (2006); Schoch et al. (2009)
*Plowrightia obietis*	ATCC 24339	N/A	EF114703	EF114727	N/A	N/A	Winton et al. (2007)
*Plowrightia periclymeni*	178096	N/A	FJ215702	FJ215709	N/A	N/A	Li and Zhuang (2009)
*Pseudoseptoria obscura*	CBS 135103	KF251219	KF251722	N/A	KF253175	KF252708	Quaedvlieg et al. (2013)
*Pseudosydowia eucalypti*	CPC 14028	GQ303296	GQ303327	N/A	N/A	N/A	Cheewangkoon et al. (2009)
*Rhizosphaera kalkhoffii*	ATCC 26605	N/A	EF114706	EF114731	N/A	N/A	Winton et al. (2007)
*Rhizosphaera oudemansii*	rhoubc	N/A	EF114707	EF114732	N/A	N/A	Winton et al. (2007)
*Rhizosphaera pini*	rhpisr	N/A	EF114708	EF114733	N/A	N/A	Winton et al. (2007)
*Stylodothis puccinioides*	CBS 193.58	MH857753	MH869286	N/A	N/A	N/A	Vu et al. (2019)
*Sydowia polyspora*	CBS 116.29	MH855019	MH866487	N/A	N/A	N/A	Vu et al. (2019)

AFTOL-ID, Assembling the Fungal Tree of Life; ATCC, American Type Culture Collection, Manassas, VA, USA; CBS, Culture collection of the Westerdijk Fungal Biodiversity Institute, the Netherlands; CPC, Culture collection of Pedro Crous, housed at CBS; FMR, Culture collection of the Faculty of Medicine at the Rovira i Virgili University, Reus, Spain; MFLU, MFLUCC, Mae Fah Luang University, Chiang Rai, Thailand; SQUCC, the Sultan Qaboos University culture collection, Oman; UAMH, UAMH Centre for Global Microfungal Biodiversity, the Dalla Lana School of Public Health at the University of Toronto; Type and reference collections are denoted with a superscripted “T”; N/A, no information available.

**Figure 1:**
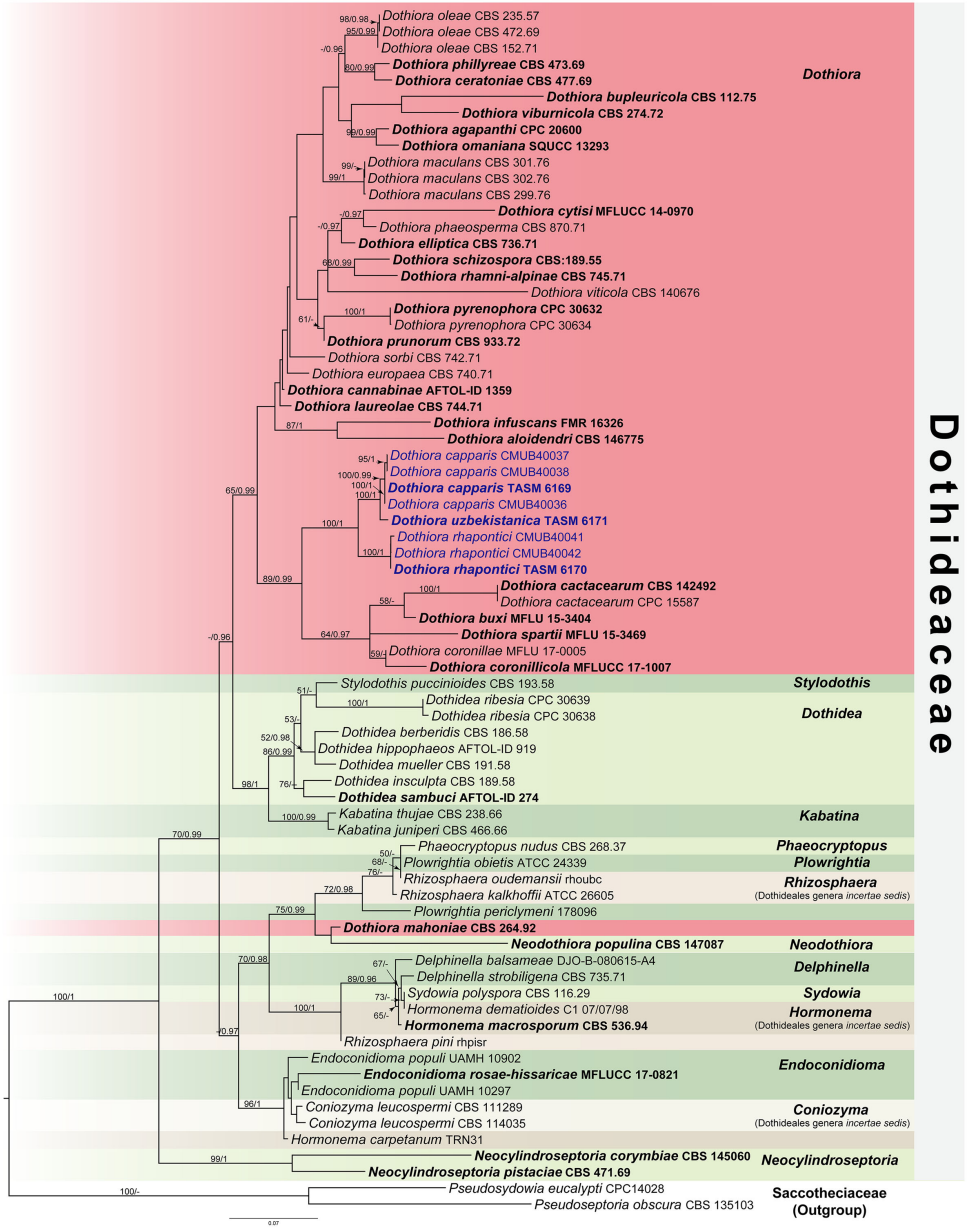
RAxML tree generated by maximum likelihood analysis of combined LSU, ITS, SSU, *TEF1*, and *TUB*2 sequence data representing Dothideaceae. Bootstrap support values for maximum likelihood (ML, left) ≥50% and Bayesian posterior probabilities (PP, right) ≥0.95 are indicated above the nodes. Hyphens (-) represent support values <50% ML/0.95 PP. The tree is rooted to *Pseudoseptoria obscura* (CBS 135103) and *Pseudosydowia eucalypti* (CPC14028). The ex-type strains are in bold, and the newly generated sequences in this study are in blue.

The ML tree was accomplished using the RAxML-HPC2 on XSEDE (v. 8.2.12) (Stamatakis, 2014) under the GTRGAMMA substitution model of nucleotide substitution with 1,000 bootstrap (BS) iterations. For BI analyses, the best-fit model of the sequence evolution of each locus was estimated using the Akaike information criterion (AIC) in MrModeltest v. 2.3 (Nylander, 2008) implemented in PAUP v. 4.0b10 (Swofford, 2002). The GTR+G+I substitution model was the best-fit model for all loci. The BI tree was executed with MrBayes v. 3.2.6 (Ronquist et al., 2012) to evaluate posterior probabilities (PP) (Rannala and Yang, 1996; Zhaxybayeva and Gogarten, 2002) by Markov Chain Monte Carlo sampling (BMCMC). Four simultaneous Markov chains were run for 5,000,000 generations, with the trees sampled every 100th generation resulting in 50,000 trees. The run was stopped when the standard deviation of split frequencies reached below 0.01. The first 12,500 trees were discarded as the burn-in phase of the analyses, while the remaining 37,500 trees were calculated for PP in the majority rule consensus tree. The resulting phylogenetic trees were drawn using FigTree v1.4.0 (Rambaut, 2016) and edited using Adobe Illustrator Version 25.2.3 and Adobe Photoshop Version 22.4.2 (Adobe Systems., U.S.A.). ML bootstrap values ≥50% and Bayesian PP ≥0.95 were placed above each node (**Figure 1**). The new nucleotide sequence data are deposited in GenBank (**Table 1**). The final alignment and tree were deposited in TreeBASE (http://www.treebase.org/) under the accession number S31239 and URL http://purl.org/phylo/treebase/phylows/study/TB2:S31239?x-access-code=12b27b6543ffd8301eed3a89eb09aed8&format=html.

## Results

### Phylogenetic analyses

The combined dataset of LSU, ITS, SSU, *TEF1*, and *TUB*2 sequence data comprises 74 taxa, which represent strains from Dothideaceae and two outgroup taxa in Saccotheciaaceae, *Pseudoseptoria obscura* Quaedvl., Verkley & Crous (CBS 135103) and *Pseudosydowia eucalypti* (Verwoerd & du Plessis) Thambug. & K.D. Hyde (CPC14028) (**Table 1**). The combined sequence alignment consisting of 4,055 characters was analyzed by ML and BI. A best scoring RAxML tree with a final likelihood value of −19,627.809539 is presented in **Figure 1**. The matrix of the combined dataset had 1,384 distinct alignment patterns and 53.70% of undetermined characters or gaps. Estimated base frequencies were A = 0.253918, C = 0.235773, G = 0.266686, T = 0.243623; substitution rates were AC = 1.637334, AG = 2.504185, AT = 1.806129, CG = 1.203612, CT = 7.247662, GT = 1.000000; and gamma distribution shape parameter α = 0.504007. Bayesian posterior probabilities (PP) from MCMC were evaluated with the final average standard deviation of split frequencies = 0.009894. The Bayesian analysis resulted in a tree with similar topology and clades as the ML tree. Phylogenetic analyses of a combined LSU, ITS, SSU, *TEF1*, and *TUB2* sequence data (**Figure 1**) show that three novel species of *Dothiora* in this study form a clade within the Dothideaceae with high support (100% ML and 1 PP) and sister to the clade containing *Dothiora buxi* Jayasiri, Camporesi & K.D. Hyde, *D. cactacearum* Crous, *D. coronillae* Dissan., Camporesi & K.D. Hyde, *D. coronilicola* Dissan., Camporesi & K.D. Hyde, and *D. spartii* Dissan., Camporesi & K.D. Hyde.

### Taxonomic descriptions

**Figure 2:**
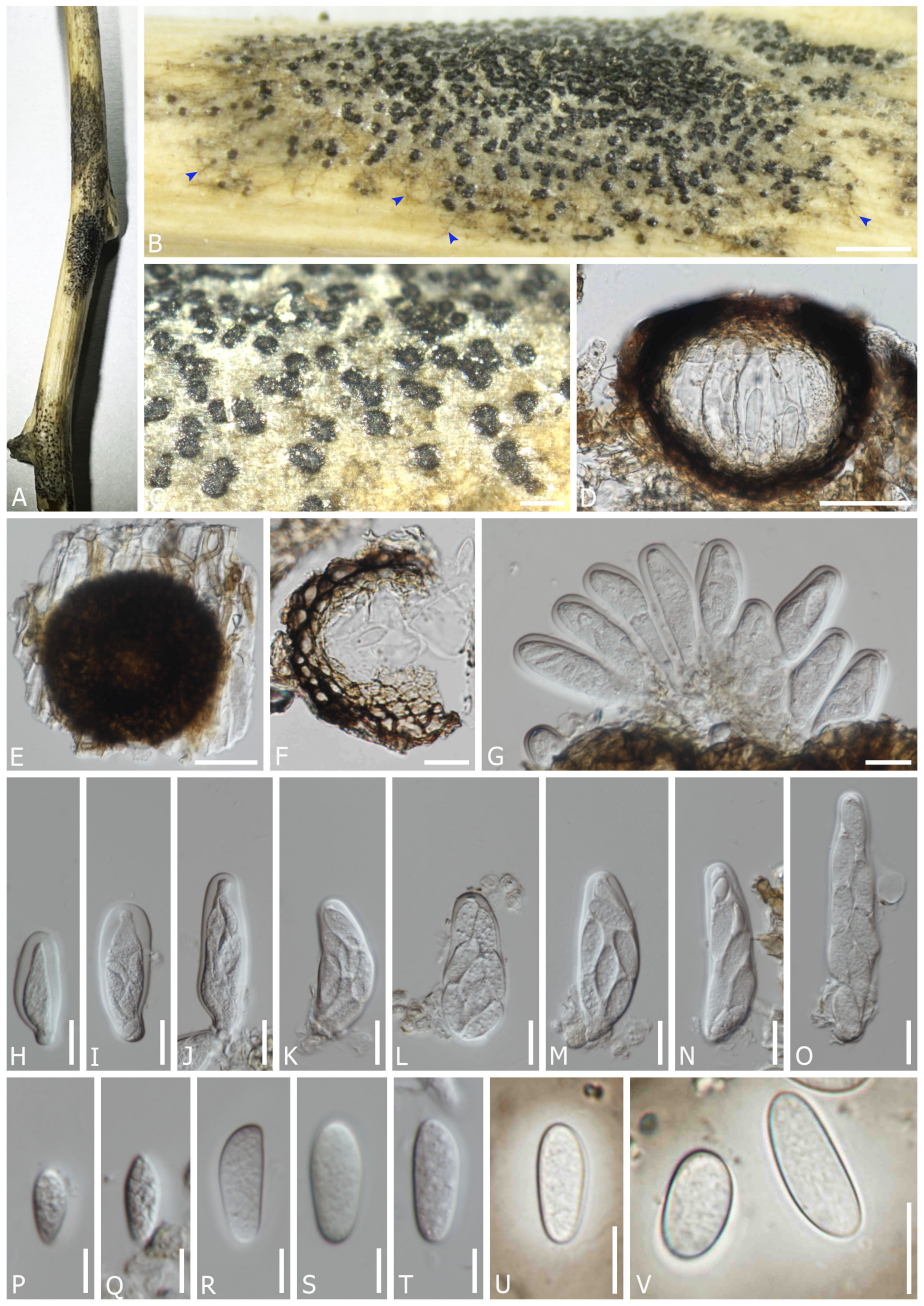
*Dothiora capparis* (TASM 6169, holotype). **(A)** Appearance of ascomata on host. **(B, C)** Fruiting bodies under a stereo microscope [superficial mycelia; blue arrows in panel **(B)**]. **(D)** Section through the ascoma. **(E)** Squash mount. **(F)** Peridium. **(G–O)** Asci. **(P–T)** Ascospores (mounted in ddH_2_O). **(U, V)** Ascospore with mucilaginous sheath (mounted in Indian ink). Scale bars: **(B)** 1000 µm, **(C)** 200 µm, **(D, E)** 50 µm, **(F–O)** 20 µm, and **(P–V)** 10 µm.

*Dothiora capparis* Senwanna, N. Suwannar., & Gafforov, sp. nov. (**Figure 2**).

MycoBank number: MB851612

Etymology: Name reflects the host genus *Capparis* from which it was isolated.

*Saprobic* on dead twigs and branches of *Capparis spinosa* L. Sexual morph: *Mycelium* partly immersed on the substrate, simple to branched, septate, smooth-walled, pale brown to brown hyphae. *Ascomata* 90–115 µm diam. × 55–100 µm high, semi-immersed to erumpent through the epidermis, solitary or clustered, scattered, globose, dark brown to black, with single locules. *Peridium* (13–)17–28(–33) µm wide, composed of cells of *textura angularis*, an outer layer dark brown to black, thick-walled, an inner layer hyaline, thin-walled. *Hamathecium* lacking pseudoparaphyses. *Asci* (47.5–)54–82(–88) × (16–)21–28(–34) µm (*x̅* = 66 × 24 µm, *n* = 40), 8-spored, bitunicate, fissitunicate, cylindro-clavate, pedicellate, apically rounded, with a small ocular chamber. *Ascospores* (15–)19–28 × (6–)9–12 µm (*x̅* = 24 × 10 µm, *n* = 50), overlapping 1–2-seriate, fusoid to ovoid, one end narrower than other, hyaline, aseptate, smooth-walled with granular contents, surrounded by a distinct mucilaginous sheath, 4–10 µm wide at sides. Asexual morph: undetermined.

Material examined: UZBEKISTAN, Jizzakh Region, Forish District, Yangiqishloq village, dead twigs and branches of *Capparis spinosa* L. (Capparaceae), 05 May 2021, Y. Gafforov, M. Yarasheva, YG-F-2-1 (TASM 6169, holotype; CMUB40035, paratype); *ibid*, *Capparis spinosa*, 05 May 2021, Y. Gafforov, M. Yarasheva, YG-F-2-2 (CMUB40036); *ibid*, Nurota District, Nurota, dead twigs and branches of *Capparis spinosa*, 07 May 2021, Y. Gafforov, YG-F-5-1 (CMUB40037); *ibid*., dead twigs of *Capparis spinosa*, 07 May 2021, Y. Gafforov, YG-F-5-2 (CMUB40038).

Notes: In a BLASTn search of NCBI GenBank, the closest match of the LSU sequence of *D. capparis* (TASM 6169, holotype) was *D. spartii* (strain MFLU 15-3469; MF443250) with 99.52% similarity; the closest match of the ITS sequence with 98.36% similarity was *D. coronill*ae (MFLU 17-0005; NR157481); the closest matches of the SSU sequence with 100% similarity were *D. pyrenophora* (CPC 30632; KY929125), *D. prunorum* (C. Dennis & Buhagiar) Crous (CBS 933.72; EU707926), and *D. cannabinae* Froid. (CBS 737.71; NG062696), respectively; while the closest matches of the *TEF1* sequence with 97.77% similarity were *D. oleae* (DC.) Crous (SAG 68856-SF; KY613610, CBS 472.69; KU728588, CBS 235.57; KU728587, and CBS 152.71; KU728586) and *D. ceratoniae* (Quaedvl., Verkley & Crous) Crous (CBS 441.75; KU728581). Based on the multi-gene phylogenetic analyses, *D. capparis* (TASM6169, CMUB40036, CMUB40037, and CMUB40038) form a distinct lineage with 100% ML and 0.99 PP statistical support (**Figure 1**) and is closely related to *D. uzbekistanica*. Albeit the phylogenetic relationships of those four strains clustering in two different clades, a comparison of ITS and *TEF1* nucleotides shows that strains TASM 6169 and CMUB40036 differ from strains CMUB40037 and CMUB40038 in 1/589 bp (0.17%) and 2/479 bp (0.42%), respectively. Moreover, *D. capparis* differs from *D. uzbekistanica* in having ascospores with a distinct mucilaginous sheath, while the latter lacks this character. A nucleotide comparison of the *TEF1* gene indicated that all strains of *D. capparis* differ from *D. uzbekistanica* by 20/414 bp (4.83%). According to Jeewon and Hyde (2016), a nucleotide comparison of reliable genes must reveal a difference of more than 1.5% to confirm the existence of a different species. Therefore, *D. capparis* and *D. uzbekistanica* are different species.

**Figure 3:**
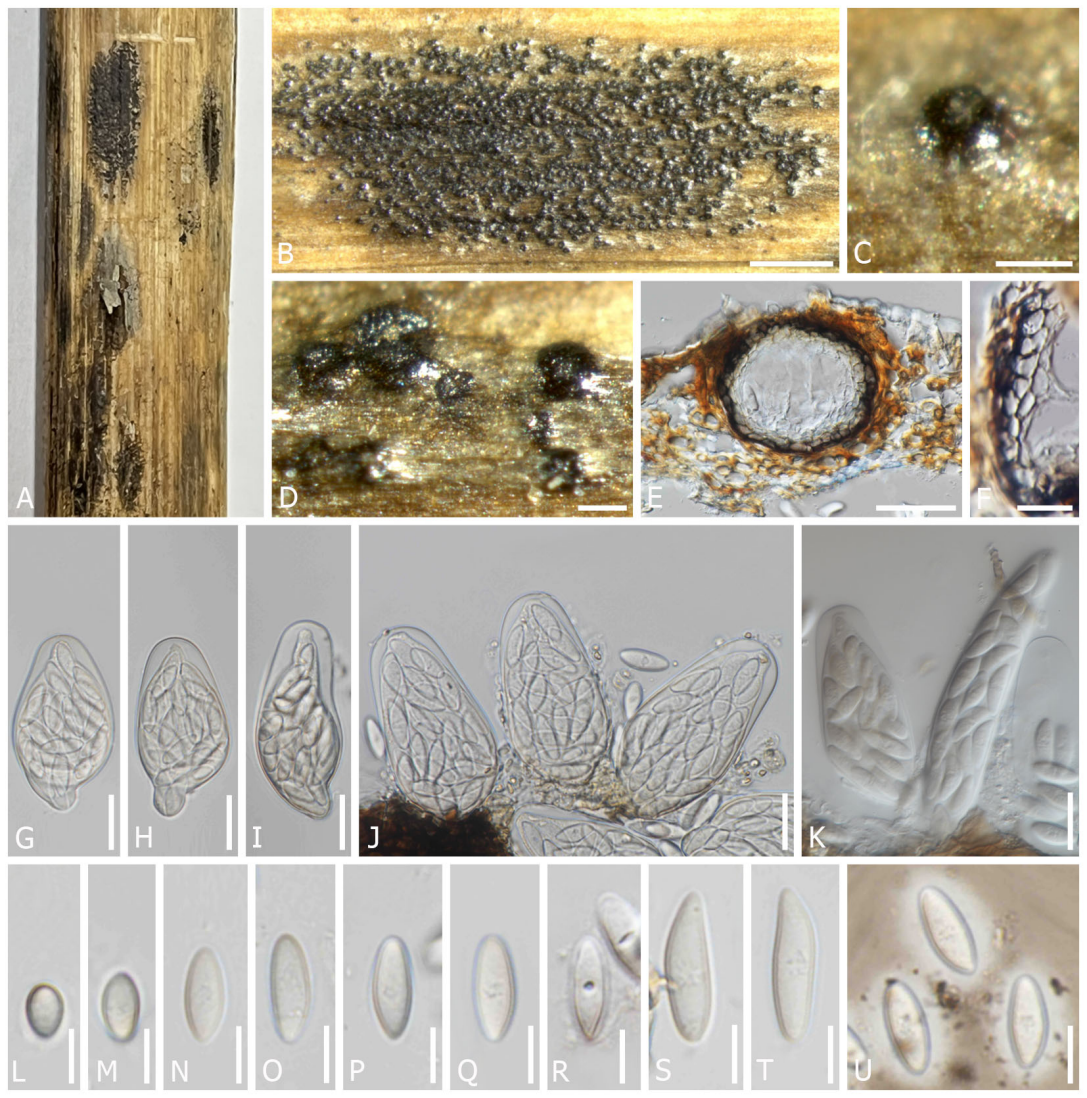
*Dothiora rhapontici* (TASM 6170, holotype). **(A)** Appearance of ascomata on host. **(B–D)** Fruiting bodies under a stereo microscope. **(E)** Section through the ascoma. **(F)** Peridium. **(G–J)** Asci (mounted in ddH_2_O). **(K)** Asci (mounted in lactophenol). **(L–T)** Ascospores (mounted in ddH_2_O). **(U)** Ascospores with mucilaginous sheath (mounted in Indian ink). Scale bars: **(B)** 1,000 µm, **(C, D)** 100 µm, **(E)** 50 µm, **(F–K)** 20 µm, and **(L–U)** 10 µm.

*Dothiora rhapontici* Senwanna, N. Suwannar., & Gafforov, sp. nov. (**Figure 3**).

MycoBank number: MB851613

Etymology: The name reflects the host genus *Rhaponticum* from where it was isolated.

*Saprobic* on dead twigs of *Rhaponticum repens* L. Sexual morph: *Ascomata* 80–155 µm diam. × 60–120 µm high, semi-immersed to erumpent through the epidermis, solitary or clustered, scattered, globose to subglobose, dark brown to black, with single locules. *Peridium* 12–21(–23) µm wide, composed of cells of *textura angularis*, an outer layer dark brown to black, thick-walled, an inner layer hyaline, thin-walled. *Hamathecium* lacking pseudoparaphyses. *Asci* 63–80(–113) × (23–)29–39 µm (*x̅* = 73 × 34 µm, *n* = 20), polysporous (24 or more spores), bitunicate, cylindro-clavate, pedicellate, apically rounded. *Ascospores* (14–)16–26(–28) × 6–7(–8) µm (*x̅* = 19 × 7 µm, *n* = 40), multi-seriate, fusoid to ovoid, one end narrower than other, hyaline, aseptate, with a central concave depression, smooth-walled with granular contents, surrounded with a mucilaginous sheath, 2–4.5 µm wide at sides.

Material examined: UZBEKISTAN, Tashkent Region, Ugam-Chatkal National Park, Chimyon, Western Tien-Shan Mountains, dead twigs of *Rhaponticum repens* (L.) Hidalgo (Asteraceae), 21 July 2019, Y. Gafforov, M. Yarasheva, YG-S-22-4 (TASM 6170, holotype; CMUB40039, paratype); Jizzakh Region, Zaamin District, Zaamin National State Park, dead branches of *Capparis spinosa* L. (Capparaceae), 27 August 2020, Y. Gafforov, A. Abdurazakov, YG-ZMB-40-1 (CMUB40040); *ibid*., dead branches of *Capparis spinosa*, 27 August 2020, Y. Gafforov, A. Abdurazakov, YG-ZMB-40-2 (CMUB 40041).

Notes: In a BLASTn search of NCBI GenBank, the closest match of the LSU sequence of *D. rhapontici* (TASM6170, holotype) was *D. cannabinae* (AFTOL-ID 1359; MF443250) with 99.66% similarity; the closest match of the ITS sequence with 98.16% similarity was *D. coronillae* (MFLU 17-0005; NR157481); the closest matches of the SSU sequence with 100% similarity were *D. pyrenophora* (CPC 30632; KY929125), *D. prunorum* (CBS 933.72; EU707926), and *D. cannabinae* (CBS 737.71; NG062696), respectively; while the closest matches of the *TEF1* sequence with 96.09% similarity was *D. phillyreae* (CBS 473.69; KU728590). The phylogenetic analysis reveals that *D. rhapontici* formed a distinct sister clade with *D. capparis* and *D. uzbekistanica*, which is statistically supported (100% ML and 1 PP) (**Figure 1**). *Dothiora rhapontici* shares similar morphological features of ascospores with related species; however, *D. rhapontici* can be distinguished from those latter species by having polysporous asci and having longer and wider ascospores.

**Figure 4:**
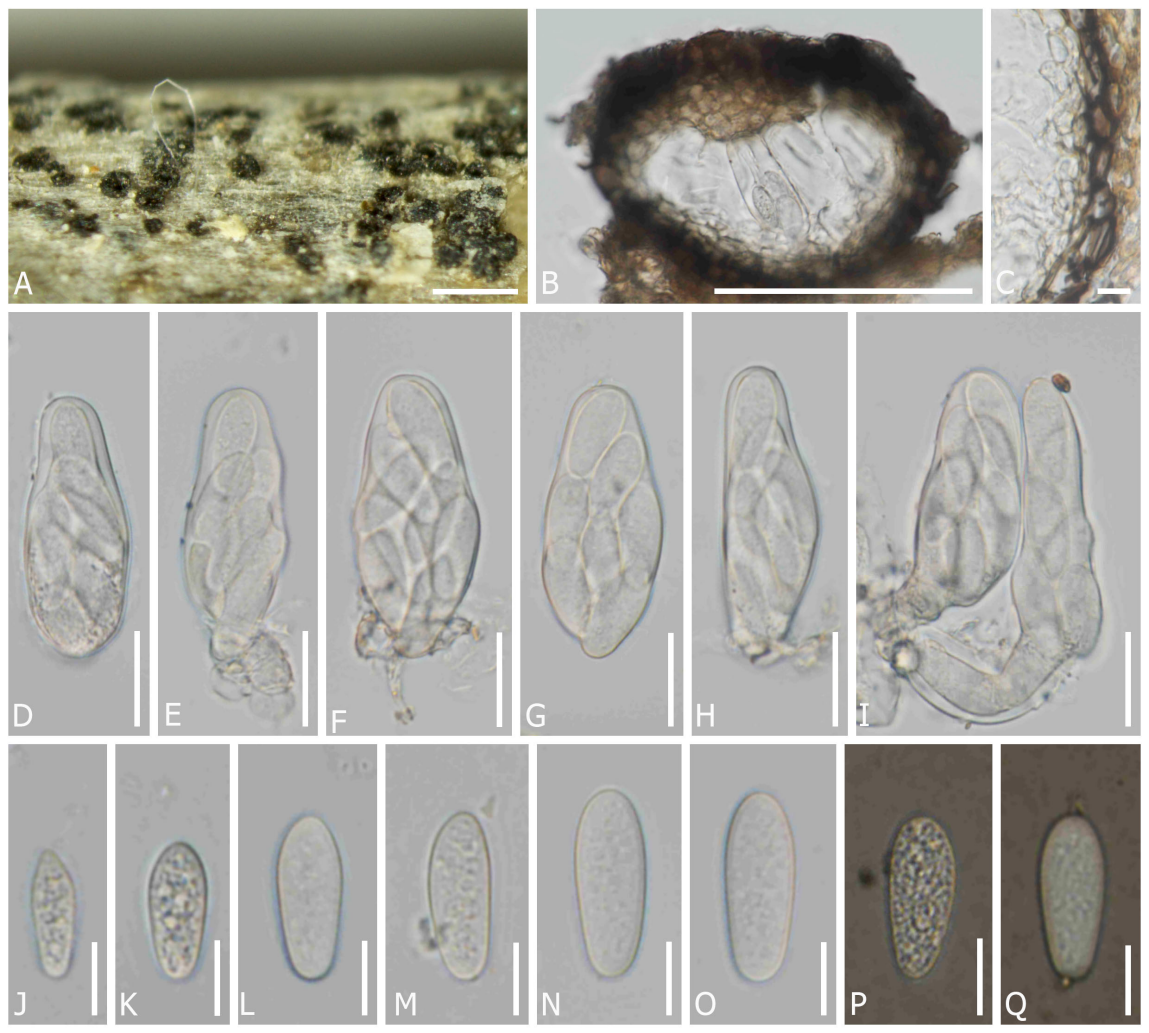
*Dothiora uzbekistanica* (TASM 6171, holotype). **(A)** Fruiting bodies under a stereo microscope. **(B)** Section through the ascoma. **(C)** Peridium. **(D–I)** Asci. **(J–O)** Ascospores (mounted in ddH_2_O). **(P, Q)** Ascospores (mounted in Indian ink). Scale bars: **(A)** 200 µm, **(B)** 100 µm, **(C, J–Q)** 10 µm, and **(D–I)** 20 µm.

*Dothiora uzbekistanica* Senwanna, N. Suwannar., & Gafforov, sp. nov. (**Figure 4**).

MycoBank number: MB851614

Etymology: The name refers to the country Uzbekistan, where it was collected.

*Saprobic* on dead twigs of unknown angiosperm perennial plants. Sexual morph: *Ascomata* 95–155 µm diam. × 80–135 µm high, semi-immersed to erumpent through the epidermis, solitary or clustered, globose to subglobose, black, with single locules. *Peridium* (10–)15–25(–30) µm wide, composed of cells of *textura angularis*, an outer layer dark brown to black, thick-walled, an inner layer hyaline, thin-walled. *Hamathecium* lacking pseudoparaphyses. *Asci* (50–)54–66(–70) × (19–)21–27 µm (*x̅* = 58 × 22 µm, *n* = 20), 8-spored, bitunicate, fissitunicate, cylindro-clavate, pedicellate, apically rounded. *Ascospore* (16–)19–26 × (5–)9–11 µm (*x̅* = 22 × 9.5 µm, *n* = 45), overlapping one to two-seriate, fusoid to ovoid, one end narrower than other, hyaline, aseptate, smooth-walled with granular contents, lacking a mucilaginous sheath. Asexual morph: undetermined.

Material examined: UZBEKISTAN, Jizzakh Region, Forish District, Yangiqishloq village, dead twigs of unknown angiosperm plants, 05 May 2021, Y. Gafforov, M. Yarasheva, YG-F-4-2 (TASM 6171, holotype; CMUB40042, paratype).

Notes: In a BLASTn search of NCBI GenBank, the closest matches of the LSU sequence of D. uzbekistanica (TASM 6171, holotype) is *D. spartii* (strain MFLU 15-3469; MF443250) with 99.52% similarity, the closest matches of the ITS sequence with 98.16% similarity, was *D. coronillae* (MFLU 17-0005; NR157481), the closest matches of the SSU sequence with 100% similarity was *D. pyrenophora* (CPC 30632; KY929125), *D. prunorum* (CBS 933.72; EU707926) and *D. cannabinae* (CBS 737.71; NG062696), respectively, while the closest matches of the TEF1 sequence with 97.21% similarity was *D. oleae* (SAG 68856-SF; KY613610, CBS 472.69; KU728588, CBS 235.57; KU728587, and CBS 152.71; KU728586) and *D. ceratoniae* (CBS 441.75; KU728581). In the phylogenetic analysis, *D. uzbekistanica* forms a distinct lineage basal to *D. capparis* with 100% ML and 1 PP bootstrap support (**Figure 1**). Moreover, the different characteristics of the microscopic features and the nucleotide comparison data of *D. uzbekistanica* differ from *D. capparis* have been mentioned above.

## Discussion

Historically, *Dothiora* has relied on morphological studies, and only a few sequences of species are available in GenBank. In this study, three novel species of *Dothiora* are introduced in the family Dothideaceae from the Central Asian region based on their morphological distinctiveness and phylogenetic analyses. Although a living culture from an isolated ascospore could not be obtained, the fungal DNA was extracted directly from the ascomata. The connection between sexual and asexual morphs is likewise unknown. The individual phylogenetic analyses of ITS or LSU separated *Dothiora* species from other genera in Dothideaceae, but their placement was otherwise unresolved (Crous and Groenewald, 2017; Crous et al., 2018a; Crous et al., 2018b). Therefore, the combination of LSU, ITS, and SSU sequence data was previously used to clarify the relationships among the species in Dothideaeceae, although there is no strong statistical support (Thambugala et al., 2014; Hyde et al., 2017; Crous et al., 2020, Crous et al., 2022; Hongsanan et al., 2020; Boonmee et al., 2021). Gao et al. (2021) recommended using a combination of the nuclear ribosomal region (ITS, LSU, and SSU) and the protein-coding gene regions (*TEF1* and *TUB2*) to clarify the relationships of Dothideaceae. Our attempts to obtain *TUB2* sequence data for our new strains were unsuccessful; however, the data from the combined sequence analyses of the ITS, LSU, SSU, and *TEF1* loci are not well-resolved for most *Dothiora* species. Thus, a phylogenetic analysis based on a combination of five loci was generated for a better phylogenetic relationship within the family and genus. Our multigene phylogeny (**Figure 1**) revealed that the generic placement within Dothideaceae, comprising 14 genera and three Dothideales genera *incertae sedis*, *viz.*, *Coniozyma*, *Hormonema*, and *Rhizosphaera*, was similar to those of Hongsanan et al. (2020) and Gao et al. (2021). Most *Dothiora* taxa clustered together in its own clade with 65% ML, 1 PP statistical support, excepting *D. mahoniae* (A.W. Ramaley) Crous (strain CBS 264.92) (**Figure 1**).

A checklist of 69 accepted *Dothiora* species, including details of each species based on recorded from Index Fungorum (2024), MycoBank (2024), and published articles, is provided in **Table 2**. *Dothiora* have a cosmopolitan distribution and are mainly saprobic, found in decaying wood and plant litter in terrestrial environments. Members of *Dothiora* have been recorded on 36 host plant families, *viz*., Aceraceae, Adoxaceae, Amaryllidaceae, Apiaceae, Apocynaceae, Araliaceae, Asphodelaceae, Asteraceae, Berberidaceae, Buxaceae, Cactaceae, Capparaceae, Caprifoliaceae, Celastraceae, Convolvulaceae, Cupressaceae, Elaeagnaceae, Ericaceae, Fabaceae, Grossulariaceae, Lythraceae, Magnoliaceae, Oleaceae, Pinaceae, Plantaginaceae, Podocarpaceae, Rhamnaceae, Rosaceae, Rubiaceae, Salicaceae, Staphyleaceae, Symplococeae, Tamaricaceae, Taxaceae, Thymelaeaceae, and Vitaceae. This study is the first record of *Dothiora* on Asteraceae and Capparaceae. The genus has mainly been reported in the USA (22 species), Italy (10 species), Canada (8 species), and Switzerland (7 species). The identification of *Dothiora* species was initially based on its sexual morph. A total of 37 species of *Dothiora* are known only for their sexual morph, 15 species are known only for their asexual morph, and another eight species have no information available. There are only eight *Dothiora* species that have asexual-sexual morph connections, *viz.*, *D. buxi*, *D. cytisi* (Wanas., Camporesi, E.B.G. Jones & K.D. Hyde) Crous, *D. lonicerae* Fuckel, *D. pyrenophora*, *D. schizospora* Luttr., *D. sorbi* (Wahlenb.) Fuckel, *D. sphaeroides* (Pers.) Fr., and *D. taxicola* (Peck) M.E. Barr. To date, 31 species have been reported based on molecular data, and only in four species have been proven the connectivity of sexual and asexual morphs through sequence data and culture studies.

**Table 2:** Morphology, host information, locality, sequence data, and related references *Dothiora* reported worldwide based on the record of Species Fungorum and MycoBank database 2024.

*Dothiora* species	Host	Locality	Morphology	Sequence data	References
*D. agapanthi*	On leaves of *Agapanthus* sp. (Amaryllidaceae)	South Africa	**Sexual**: Undetermined **Asexual**: *Conidiomata* pycnidial, globose with long neck, brown, to 250 μm diam, with central ostiole, exuding a creamy conidial mass; *Conidiophores* hyaline, smooth, ampulliform to doliiform, 5–7 × 5–6 μm, with central phialidic locus; *Conidia* hyaline, smooth, guttulate, subcylindrical, apex obtuse, tapering to a truncate hilum (8–)10–12(–13) × 3(–3.5) μm; *Hyphae* becoming brown, verruculose, and constricted at septa, giving rise to a Hormonema-like synasexual morph.	Available	Crous and Groenewald (2016)
*D. aloidendri*	On leaves of *Aloidendron dichotomum* (Asphodelaceae)	South Africa	**Sexual**: Undetermined **Asexual**: *Conidiomata* pycnidial, globose, black, glabrous, 200–350 µm diam, aggregated in dense clusters, exuding a creamy conidial mass; *Conidiophores* reduced to conidiogenous cells lining the inner cavity, hyaline, smooth, ampulliform to doliiform, phialidic, 6–9 × 5–7 µm; *Conidia* solitary, straight, subcylindrical, aseptate, guttulate, hyaline, smooth, thin-walled, apex obtuse, tapering at base to truncate hilum, 1–1.5 µm diam, (10–)12–13(–14) × (3–)4 µm.	Available	Crous et al. (2020)
*D. amelanchieris*	In stems of *Amelanchier alnifolia* (Rosaceae)	Canada	**Sexual**: *Ascostromata* up to 500 μm wide, 300 μm high; Hamathecium lacking pseudoparaphyses; *Asci* 8-spored, bitunicate, fissitunicate, oblong, parallel from basal subhymenial layer, 75–90 × 12–15 μm; *Ascospores* hyaline, ellipsoid to somewhat obovoid, 20–30(–32) × 7–10 μm, 5–7 transversely septate, one longitudinal septum in one, two, or three of mid cells, hemispores somewhat unequal, upper longer, constricted at first-formed septum. **Asexual**: Undetermined	N/A	Barr (2001)
*D. bupleuricola*	On leaf spot of *Bupleurum fruticosum* (Apiaceae)	France	**Sexual**: Undetermined **Asexual**: *Conidiomata* pycnidial, globose with long neck, brown, to 250 μm diam, with central ostiole, exuding a creamy conidial mass; *Conidiophores* hyaline, smooth, globose to allantoid 5–7 × 4–6 μm, with central phialidic locus. *Conidia* hyaline, smooth, guttulate, subcylindrical, apex obtuse, tapering to a truncate hilum (8–)9–10(–12) × 2(–2.5) μm.	Available	Crous and Groenewald (2016)
*D. buxi*	On dead branch, dying leaves and twigs (hemibiotrophic) of *Buxus sempervirens* (Buxaceae)	Italy and Russia	**Sexual**: *Ascostromata* 500–1000 μm long, 220–250 μm high, 320–340 μm diam., erumpent through the epidermis, solitary or clustered, globose, brown to black, with single locules, with a central longitudinal slit-like opening. *Peridium* 32–83 μm wide; *Hamathecium* lacking pseudoparaphyses. *Asci* 100–115 × 14–21 μm, 32-spored, bitunicate, fissitunicate, cylindro-clavate, short pedicellate, apically rounded, with a small ocular chamber. *Ascospores* 11–15 × 5.4–7 μm, bi-seriate to multi-seriate, hyaline to very pale brown, aseptate, fusoid to ovoid, one end narrower than other, smooth-walled with granular contents, with a thin mucilaginous sheath. **Asexual**: *Conidiomata* 182–263 μm high × 383–447 μm diam., pycnidial, globose to subglobose, visible as brown to black, pustulate on the lower leaf surface; Peridium 21–43 lm wide; *Conidiogenous cells* 11–17 × 6–11 μm diam., phialidic, subglobose, hyaline. *Conidia* 10–16 × 6–19 μm diam., ellipsoid to obovoid, rounded at top, narrow at base, guttulate, smooth, 1-celled, hyaline.	Available	Hyde et al. (2016); Tibpromma et al. (2017)
*D. cactacearum*	On phyllodes of Cactaceae	USA (Texas)	**Sexual**: Undetermined **Asexual**: *Conidiomata* separate, erumpent, pycnidial, globose, medium brown, 150–300 μm diam., with a central ostiole, exuding a creamy conidial mass; *Conidiophores* reduced to conidiogenous cells lining the inner cavity, hyaline, smooth, ampulliform to doliiform, 7–15 × 7–15 μm, phialidic, at times with percurrent proliferation and prominent collarette; *Conidia* hyaline, smooth, guttulate, subcylindrical to broadly ellipsoidal, apex obtuse, tapering to a truncate, protuding hilum, 2–3 μm diam., (12–)14–17(–19) × (5–)6–7.5(–8) μm, conidia becoming brown, verruculose and constricted at the septa.	Available	Crous and Groenewald (2016)
*D. cannabinae*	On dead branches of *Daphne cannabina* (Thymelaeaceae)	India	N/A	Available	Froidevaux (1972)
*D. ceratoniae*	On dead leaves of *Nerium oleander* (Apocynaceae), on dead leaves of *Arbutus unedo* (Ericaceae), and on leaves of *Ceratonia siliqua* (Fabaceae)	Italy and Spain	**Sexual**: Undetermined **Asexual**: *Conidiomata* brown, cupulate, short-stipitate, rim up to 300 µm diam, 100–180 µm tall, tapering toward base, 20–50 µm diam; *Conidiogenous cells* hyaline, smooth, ampulliform, 7–12 × 4–6 µm; apex 2 µm diam, with prominent periclinal thickening; *Conidia* hyaline, smooth, granular or not, cylindrical with obtuse apex, tapering at base to truncate scar 1 µm diam, aseptate, (10–)12–14(–16) × 3(–3.5) µm.	Available	Quaedvlieg et al. (2013); Crous and Groenewald (2016)
*D. coronillae*	On dead aerial branches of *Coronilla emerus* (Fabaceae)	Italy	**Sexual**: *Ascostromata* 185–310 μm high × 220–250 μm diam., immersed or erumpent through the epidermis, solitary or clustered, globose, brown to black, with single locules; *Peridium* 32–83 μm wide; *Asci* 60–110 × 14–21 μm, 8-spored, bitunicate, fissitunicate, cylindro-clavate, short pedicellate, apically rounded, with a small ocular chamber; *Ascospores* 17–22 × 7–9 μm, bi-seriate to multi-seriate, hyaline, aseptate, fusoid to ovoid, one end narrower than other, smooth-walled with granular contents, lacking a mucilaginous sheath. **Asexual**: Undetermined	Available	Hyde et al. (2017)
*D. coronillicola*	On dead branches of *Coronilla emerus* (Fabaceae)	Italy	**Sexual**: *Ascomata* 215–430 × 240–285 µm, immersed or erumpent through the epidermis, solitary or clustered, globose, brown to black, with single locules; *Peridium* 39–76 µm wide; *Asci* 80–145 × 15–30 µm, 8-spored, bitunicate, fissitunicate, cylindro-clavate, pedicellate, apically rounded, with a small ocular chamber; *Ascospores* 21–25 × 8–11 µm, bi-seriate to multiseriate, hyaline, aseptate, fusoid to ovoid, one end narrower than the other, smooth-walled with granular contents, lacking a mucilaginous sheath. **Asexual**: Undetermined	Available	Boonmee et al. (2021)
*D. cytisi*	On dead and hanging branches of *Cytisus scoparius* (Fabaceae)	Italy	**Sexual**: *Ascostromata* 180–250 × 170–210 μm, superficial, semi-immersed to erumpent, solitary, scattered, broadly oblong, dark brown to black, coriaceous, uniloculate; *Peridium* 35–45μm wide at the base, 30–40μm wide at the sides; *Hamathecium* lacking pseudoparaphyses; *Asci* 70–90× 20–30 μm, 8-spored, bitunicate, fissitunicate, clavate to broadly-clavate, short pedicellate, thickened and rounded at apex, with an ocular chamber; *Ascospores* 25–35 × 7–10μm, overlapping 1–2-seriate, hyaline, broadly fusiform, rounded at both ends, 1-septate, with a median septum, constricted at the septum, smooth-walled, lacking a mucilaginous sheath. **Asexual**: *Conidiomata* stromatic, immersed in agar to superficial, uni- to multi-loculate, globose to subglobose, glabrous, ostiole central, with minute papilla; *Conidiomata walls* composed of several layers of hyaline to dark brown, pseudoparenchymatous cells, organized in a *textura angularis*; *Conidiophores* arising from basal cavity of conidiomata, mostly reduced to conidiogenous cells; *Conidiogenous cells* holoblastic, phialidic, discrete, ampulliform to cylindric-clavate, hyaline, aseptate, smooth-walled; *Conidia* 25–35 × 6–9 μm, solitary, 1-celled, fusiform to falcate, with narrowed ends, initially hyaline, becoming pale brown at maturity, aseptate, smooth and thin-walled, guttulate, contents granular.	Available	Li et al. (2016)
*D. dothideoides* ^2^	*Prunus emarginata* (Rosaceae), On dead twigs of *Shepherdia canadensis* (Elaeagnaceae) and on dead branch of *Populus tremuloides* (Salicaceae)	Canada and USA	**Sexual**: *Ascostromata* connected by and seated in dense subiculum; *Asci* polysporous; *Ascospores* (13–)16–18(–21) × 4–8 μm, hyaline, muriform. **Asexual**: Undetermined	N/A	Barr (2001)
*D. elegans* ^2^	On twig of *Pinus sylvestris* (Pinaceae)	USA	**Sexual**: Undetermined **Asexual**: Punctiform; *Spores* fenestrated, 25 μm long.	N/A	Saccardo (1889)
*D. elliptica*	On dry branches of *Vaccinium uliginosum* (Ericaceae) and on dead twigs of *Linaria vulgaris* (Plantaginaceae)	France and Switzerland	**Sexual**: *Ascomata* arranged in parallel, elliptical (hysteriiform), 1 mm. long, *Asci* 8-spored, elongate, 96 × 12 μm; *Ascospores* distichis, oblong-fusiform, 3-septate, hyaline, 16 × 7 μm. **Asexual**: Undetermined	Available	Fuckel (1873)
*D. ellisii* ^2^	On decayed needles of *Pinus contorta* var. *latifolia* (Pinaceae)	USA	**Sexual**: *Ascomata* compound to clustered in groups of 3–10, immersed becoming erumpent, appearing superficial, globose, depressed, glabrous, 100–150 μm wide, 150–200 μm high; *Beak* short, truncated-conical, terete, 20–25 μm long, 60–70 μm wide, composed of 2 or 3 layers of brown polygonal 7–10 × 6–8 μm cells around a 20–25 μm diam. Ostiole, without periphyses or surface setae; *Wall* in longitudinal section uniform in thickness, 15–20 μm thick, of 2 or 3 layers of 6–10 × 4–5 μm polygonal to rectangular thin-walled brown cells; *Hamathecium* not seen; *Asci* numerous, from a central base, bitunicate, broadly fusiform, 70–85 × 25–30 μm, with 8 tetraseriate ascospores; *Ascospores* broadly fusiform to obovoid, straight, 3-septate, in sequence 2:1:2, second cell from apex enlarged, first septum complete and slightly constricted, median (0.50), not constricted at additional full septa, brown, smooth, guttulate, with a conspicuous sharply delimited sheath constricted at first septum, 1.5–2 μm wide, or wider if exposed to water for a long time. **Asexual**: Undetermined	N/A	Shoemaker and Babcock (1987)
*D. eunomia*	In dry branches of *Fraxinus excelsior* (Oleaceae)	Finland	**Sexual**: *Stromata* gregarious, covered with raised fissured periderm, usually rounded, unicellular, 0.3–0.5 mm wide; *Spermatia* elongate, straight or gently curved, elongate, 4–5 μm long, 0.5–1 μm thick. **Asexual**: Undetermined	N/A	Karsten (1884)
*D. europaea*	*Alnus* sp., on dead branches of *Salix elvetica* (Salicaceae)	Switzerland and USA	**Sexual**: *Asci* polysporous; *Ascospores* hyaline, 20–25 × 5–6 μm, upper hemispore shorter and wider than lower. **Asexual**: Undetermined	Available	Barr (2001)
*D. harknessii* ^2^	On dead stems of *Convolvulus californicus* (Convolvulaceae)	USA	**Sexual**: *Stromata* immersed, visible and slightly erumpent through splits in periderm, dull black, elongate oblong or ± rounded, 1 mm or more long, up to 550 μm wide, 275–300 μm high, apex plane or shallowly depressed; *Locule* single, up to 385 μm wide, or two and ± 180 μm wide, approximately 100 μm deep; *Walls* pseudoparenchymatous, cells thick walled, dark reddish brown externally, slightly tinged reddish or hyaline internally, base 80–100 μm deep, sides 30–40 μm wide, subhymenium small celled, hyaline, in a low arching cushion; *Asci* densely packed in broad fascicle from cushion, bitunicate, 60–80 × 8–11 μm, 8-spored; interthecial tissues sparse, visible at sides and above asci; *Ascospores* 13–16 × 5–7 μm, hyaline, or slightly reddish-brown in age, obovoid, asymmetric, broader and usually longer above, 3–5-septate, constricted at submedian primary septum, longitudinal septum in mid cells, at times obliquely in one end cell, smooth-walled, contents minutely guttulate, overlapping biseriate or partially uniseriate in the ascus. **Asexual**: Undetermined	N/A	Barr (1981)
*D. hederae*	On dead branches of *Hedera helix* (Araliaceae)	Switzerland	N/A	N/A	Index Fungorum (2024)
*D. hurae*	N/A	N/A	N/A	N/A	Index Fungorum (2024)
*D. infuscans*	Isolated from the blackened wall of an industrial warehouse	Spain	**Sexual**: Undetermined **Asexual**: *Mycelium* composed of subhyaline, smooth-, thin-walled, septate hyphae, 5–7 μm wide, later becoming thick-walled, increasing the number of septa and the volume of their cells to give them a moniliform appearance, and finally the hyphae turn dark brown and produce chains of holothallic (chlamydospore-like) conidia of up to 20 μm diam, which also develop longitudinal/oblique secondary septa over time, giving consequently a ‘muriform’ aspect to these propagules; *Conidiophores* micronematous, reduced to conidiogenous cells, mostly intercalary, producing conidia on lateral, short to long conic-truncate denticles, with 1–3 per conidiogenous cell; *Conidia* holoblastic, solitary, but attached to one another by a mucilaginous substance, mostly aseptate, smooth- and thinto thick-walled, hyaline, becoming dark brown, thick-walled, roughened and mostly 1-septate, occasionally 2–3-septate, globose, ellipsoid or irregularly-shaped, prominently constricted at septa when old, unicellular conidia 8–9 × 4–5 μm, 2-celled conidia 10–13 × 6–7 μm, multi-celled conidia 18–19 × 5–7 μm; *Microcyclic conidia* produced by budding of the hyaline or pigmented conidia, solitary or in chains of up to 5 elements on inconspicuous denticles when the conidiogenous cell is young, but on protruding conical-truncate denticles when old, at one or both ends but also laterally, being smaller than the primary conidia.	Available	Crous et al. (2018a)
*D. laureolae*	On dead branches of *Daphne laureola* (Thymelaeaceae)	Italy (Sicilia)	N/A	Available	Index Fungorum (2024)
*D. lepargyrea* ^2^	On dead twigs of *Shepherdia canadensis* (Elaeagnaceae)	USA	**Sexual**: *Ascomata* 330–440 μm diam., 275–330 μm high; *Wall* 52–65 μm thick at sides, up to 104 μm thick below*; Asci* 90–104 × 15.5–20 μm, more than 32-spored; *Ascospores* 14–18(–22) × 3.5–5 μm, hyaline, obovate, broadest above and tapered to the pointed base, (1–)3(4–7)-septate, with vertical septum in one or more of mid cells **Asexual**: Undetermined	N/A	Barr (1972); Barr, (2001)
*D. lonicerae* ^1^	On dead corticate branches of *Lonicera alpigena* (Caprifoliaceae)	France and Germany	**Sexual**: *Ascomata* scattered, oblong or rounded, creeping flat on the edge; *Asci* ample, tapering toward the base, 8-spored, 112 × 18 μm; *Ascospores* disordered, irregularly oblong, obtuse on both sides, muriform, 6–8-septate, constricted at the septa, hyaline, 30–90 × 8–10 μm. **Asexual**: *Perithecia* spurious, scattered, acicular, sharp, black, with a white globule, very minute at the terminal; *Stylospores* very narrow, fusiform, curved, 4-guttulate, 32 × 2 μm, hyaline.	N/A	Fuckel (1870)
*D. maculans*	Leaf of *Acer pseudoplatanus* (Aceraceae), leaf litter of *Populus tremuloides* (Salicaceae)	Canada and Netherlands	**Sexual**: Undetermined **Asexual**: *Conidiomata* pycnidial, with central ostiole; *Conidiogenous cells* 1–2 loci, aggregated in pseudochains, encased in a thick, persistent mucoid layer; *Conidia* hyaline, smooth, subcylindrical to oblong, guttulate, apex obtuse, tapering to a truncate hilum (7–)10–12(–13) × (2.5–)3(–3.5) μm; *Hormonema-like synasexual morph* with ampulliform to doliiform, phialidic conidiogenous cells, 5–7 × 5–6 μm.	Available	Crous and Groenewald (2016)
*D. mahoniae*	On leaves of *Mahonia repens* (Berberidaceae)	USA	**Sexual**: Undetermined **Asexual**: *Mycelium* immersed, grey-black, branched, septate, 3–12 μm wide; *Conidiomata* acervular, somewhat pulvinate, erumpent, amphigenous but mostly hypophyllous, subepidermal to epidermal, separate or confluent, formed of gray-black rather thick-walled *textura angularis* at the base, changeing to brown at the level of the conidiophores, 50–400 μm diam.; *Dehiscence* by irregular rupture of the cuticle and epidermis; *Conidial masses* white to sordid; *Conidiophores* smooth, brown, lighter colored toward the apex, septate, branched at any level along the length, up to 65 μm long; *Conidiogenous cells* integrated, pale brown, smooth, cylindrical to subclavate, periclinal thickening absent, collarette absent, 6–19 × 3–6 μm; *Conidial ontogeny* holoblastic by apical wall building, maturation synchronous with conidial ontogeny, conidiogenous cell proliferation enteroblastic to produce additional conidia at ca same level or rarely, conidiogenous cell proliferation enteroblastic to produce additional conidia at successively higher levels; *Conidia* acrogenous, hyaline, aseptate, cylindrical-ellipsoidal, smooth, thin-walled, apex and base bluntly rounded or the base truncate at the indistinct scar, 6.5–12.5 × 2.5–3.5 μm.	Available	Ramaley (1992)
*D. meynae*	On dead branches of *Meyna* sp. (Rubiaceae)	India	**Sexual**: *Stroma* solitary or gregarious, dark brown to black, innate to widely erumpent through the bark, surrounded by vertically bent, more or less lobate edges of peridermis on lateral sides 1,000–1,500 μm long and 500–800 μm broad; *Asci* oblong to cylindrical, thick-walled, bitunicate, stipitate, rounded at the apex, parallel, densely clustered in one or a few locules of indefinite shape in the stroma 80–140 × 14–20 μm, situated on the thin walled hyaline tissue of isodiametric cells; *Ascospores* fusoid, muriform, 34–40 × 6–9 μm, 6–7 transverse septa, 1 or 2 incomplete longitudinal septa through one or two or few of the thickest cells, distinctly constricted in the middle, thickest slightly above the middle septum, hyaline. **Asexual**: Undetermined	N/A	Rao (1971)
*D. moravica*	On dead stems of *Evonymus europaeus* (Celastraceae)	Czech Republic	**Sexual**: *Stromata* covered by the usually evenly black or black-brown colored, often sheer shiny epidermis, not breaking out, often several densely packed together and then more or less fused together, cushion-shaped from a rounded to elliptical base, with a fairly flat apex, usually 200–300 μm in diameter, 100–200 μm high, completely closed when young, tearing quite irregularly at the apex when ripe; *Hamathecium* not seen; *Asci* polysporous, 80–120 × 18–24 μm, sessile, broadly rounded at the top, downwards only slightly tapered; *Ascospores* elongated spindle-shaped or almost club-shaped, usually with 3, rarely 5 transverse walls, usually strong in the middle, barely constricted on the remaining transverse walls, in 1–2 of the middle cells later with 1 longitudinal wall, straight or slightly curved, hyaline, usually only at the top, more tapered downwards, bluntly rounded at both ends, 15–24 × 4.5–7 μm. **Asexual**: Undetermined	N/A	Petrak (1919)
*D. oleae*	On fallen leaf, leaves and rotting fruit of *Olea europaea* (Oleaceae), *Olea cuspidate*, and *Olea* sp.	Greece, India, Israel, Italy, Pakistan, Spain, Turkey, and USA	**Sexual**: Undetermined **Asexual**: Foliicolous. *Mycelium* immersed, composed of branched, septate, pale brown to dark brown, thin- or thick-walled, smooth hyphae; *Conidiomata* pycnidial, globose, subcylindrical, or flattened at the base, dark brown, black, unilocular, 100–250 µm diam., up to 350 µm high; Ostiole single, central, circular, often becoming wide late in development; *Conidiophores* absent or poorly developed; *Conidiogenous cells* discrete, determinate, terminal or intercalary within conidiophores, phialidic, ampulliform to lageniform or subcylindrical, hyaline, smooth, thin-walled, channel and collarette minute, occasionally with a percurrent proliferation, 8–15 × 3–5 µm; *Conidia* hyaline, aseptate, cylindrical, apex obtuse, base truncate, thin-walled, smooth, with several large guttules, 15–23 × 3–5 µm.	Available	Duan et al. (2007); Crous and Groenewald (2016)
*D. omaniana*	Leaf spot on leaves of *Punica granatum* (Lythraceae)	Oman	**Sexual**: Undetermined **Asexual**: *Conidiomata* pycnidial, to 250 μm diam; *Conidiophores* hyaline, smooth, aseptate, ampulliform 4–10 × 3–4 μm, with central phialidic locus; *Conidia* solitary, hyaline, smooth, subcylindrical to oblong, guttulate, apex obtuse, aseptate, (4–)6–8(–9) × 2–4 μm; *Hyphae* brown, verruculose, and constricted at septa.	Available	Hyde et al. (2020)
*D. petrakiana* ^2^	On dry, hanging branches of *Crataegus oxyacantha* (Rosaceae)	Germany	**Sexual**: *Ascomata* scattered or gregarious, circular or elliptical, approximately 300–600 μm long, 200–400 μm wide, 150–250 μm thick or approximately 250–400 μm diam., completely closed, breaking off from the center of the apex when mature, finally irregularly and more or less widely open; *Wall* 30–40 μm at sides, 50–60 μm at the top, rarely up to 70 μm thick; *Asci* quite numerous, arranged more or less parallel, cylindraceo-clavate, anteriorly broadly rounded, posteriorly often a little saccate and contracted into a short, thickly nodular stalk, thickly sheathed, 8-spored, p. sp. 70–90 μm, rarely up to 110 μm long, 12–20 μm thick; *Ascospores* more or less di-rarely indistinctly tristic, fusoid or inequilateral, rarely curved, hyaline, 7–rarely 5–6-septate, pocket in the middle often divided by a longitudinal septum, around the middle a little but usually distinctly, otherwise not or very gently constricted, 20–30 μm rarely up to 32 μm long, 6–10 μm wide; paraphysoids few, fibrous, late muscular. **Asexual**: Undetermined	N/A	Petrak (1957)
*D. phaeosperma*	On dead branches of *Lonicera coerulea* (Caprifoliaceae)	Switzerland	N/A	Available	Froidevaux (1972)
*D. phillyreae*	On leaf litter of *Phillyrea angustifolia* (Oleaceae)	Spain (Baleares)	**Sexual**: Undetermined **Asexual**: *Conidiomata* solitary or aggregated in a stroma, brown, to 300 μm diam, with central ostiole; *Conidiogenous cells* hyaline, smooth, aseptate, ampulliform to broadly ellipsoid or doliiform, 5–10 × 5–7 μm, holoblastic with apical locus, inconspicuously phialidic; *Conidia* hyaline, smooth, subcylindrical to oblong, guttulate, apex obtuse, tapering to a truncate hilum, 1 μm diam (8–)10–11(–12) × (2.5–)3(–3.5) μm; *Hormonema-like asexual morph* with hyphae becoming brown, verruculose, constricted at septa; *Chlamydospore-like cells* up to 8 μm diam, older conidia become brown and verruculose, up to 15 μm long, 5 μm diam.	Available	Crous and Groenewald (2016)
*D. pinacea*	On fallen branch of *Pinus sylvestris* (Pinaceae)	Czech Republic	N/A	N/A	MycoBank (2024)
*D. platyasca* ^2^	In bark	USA (Albama)	**Sexual**: *Ascomata* aggregated, first with a white margin, then flat and black-margined; *Asci* numerous, ovate or subspheroid; *Ascopsores* oblong-ovate, muriform, colored, 20 × 12 μm. **Asexual**: Undetermined	N/A	Saccardo (1889)
*D. polyspora*	On branch of *Populus tremuloides* var. *aurea* (Salicaceae), and *Salix* spp. (Salicaceae)	USA (Colorado)	**Sexual**: *Ascomata* innate-erumpent, depressed-pulvinate, circular or irregular in outline, densely gregarious, smooth, black; *Locules* single or occasionally several, thick lenticular, astmomous; *Asci* polysporous (24 or more spores), cylindric-clavate, short stipitate, 90–115 × 12–15 μm, aparaphysate; *Ascospores* when mature muriform with 3 transverse septa and frequently 1 or rarely 2 longitudinal septa in the upper cells, clavate, constricted in the middle, upper half broader, hyaline, 15–18 × 5–6 μm. **Asexual**: In culture; *Conidia* hyaline, 1-celled, 8–15 × 4–6 μm.	N/A	Shear and Davidson (1940)
*D. pruni* ^2^	*Prunus emarginata* (Rosaceae), on branches of*. virginiana* var. *demissa*, and *P. virginiana* var. *melanocarpa*	Canada and USA	**Sexual**: *Ascomata* 385–660 μm diam., 275–440 μm high; *Wall* 78–104(–195) μm thick; Asci 104–140 × 15–23.5 μm, more than 32-spored (up to 42 counted); *Ascospores* 13–18 × 5–6 μm, hyaline, obovate, straight, inequilateral or somewhat bent, broadest above and tapered to the pointed base, 3–(4–5)-septate, constricted at primary septum, with vertical septum in one or both mid cells and often in upper end cell. **Asexual**: Undetermined	N/A	Barr (1972)
*D. pruni-padi*	On dead branches of *Prunus padus* (Rosaceae)	Switzerland	N/A	N/A	Index Fungorum (2024)
*D. prunorum*	On fruit of *Prunus domestica* (Rosaceae)	UK	**Sexual**: Undetermined. **Asexual**: *Young hyphae* were 3.5–8 μm diam, while older hyphae, having shorter, fatter, thicker-walled cells, were up to 13.5 μm diam. More or less cylindrical phialoconidia (8–20 × 3.3–5 μm) were produced on minute phialides. Several phialoconidia were often produced at one point on the hyphal cell (multiple phialoconidia). Secondary buds on the phialoconidia and chlamydospores were rarely seen. Arthroconidia and endospores were not observed. *Yeast-like* multiplication of the strains occurred by polar budding of single cells (4–12.5 × 1.5–4 μm) or by phialoconidial formation on centrally constricted septate cells (6.5–14 × 3–6 μm). Polar and lateral phialoconidia were also produced on cells with several septa.	Available	Dennis and Buhagiar (1973); Crous and Groenewald (2016)
*D. pyrenophora* ^1^	On dead branches of *Sorbus* sp. (Rosaceae) and on twig of *Sorbus aucuparia* (Rosaceae)	Germany and Sweden	**Sexual**: *Ascostromata* solitary to aggregated, black, immersed to erumpent, unilocular, to 400 μm diam, elliptical, pulvinate, opening by an irregular pore, upper layer dissolving with age; *Asci* bitunicate, hyaline, oblong to subcylindrical, short stipitate, 8-spored, with apiculus, 2–3 diam., 90–120 × 14–17 μm; *Ascospores* bi- to triseriate in ascus, hyaline, smooth, at times turning yellow-brown with age, fusiform, inequilateral, slightly curved, with prominent mucoid sheath when young (in water), dissolving at maturity, (5–)5(–8) transversely septate, prominently constricted at primary septum, with oblique or vertical septa in central cells, (22–)25–30(–35) × 7–8 μm, ascospores directly giving rise to asexual morph via budding, with ascomata transforming with age into large conidiomata, with apical opening completely dissolving. **Asexual**: *Conidiomata* immersed to erumpent, pycnidial, black, globose, to 300 μm diam., separate or gregarious, unilocular; *Conidiophores* lining the inner cavity, reduced to conidiogenous cells; *Conidiogenous cells* hyaline, smooth, doliiform to ampuliform, 4–9 × 4–6 μm, with minute periclinal thickening at apex; *Conidia* solitary, aseptate, hyaline, smooth, ovate to ellipsoidal, with minute guttules, subobtuse apex, truncate hilum, (5–)7–8(–9) × 3–4 μm.	Available	Fries (1849); Crous and Groenewald (2017)
*D. rhamni*	On branches of *Rhamni frangulæ*	Denmark	**Sexual**: *Ascomata* usually erupting in longitudinal series, at first orbicular or elongate, flattened, then umbilical, broad-margined, darkly open, with a rugose margin, black, disc concolorous, necleo dirty white, continuous; *Asci* cylindrical, sessile, usually curved, 8-spored, 112 × 12 μm; *Ascospores* obliquely monostrichous, oblong, attenuated on both sides, strongly constricted in the middle, then equally double, 3-septate and 1–2 longitudinal, pale colored, 15 × 7 μm; *Macrostylospores* which are sometimes present, broad-oblong, greatly attenuated on both sides, but obtuse, 4–5-septate and muriform, fucus, 20 × 9 μm; paraphyses simple, filiform, multiguttulates. **Asexual**: Undetermined	N/A	Saccardo (1889)
*D. rhamni-alpinae*	On dead branches of *Rhamnus alpina* (Rhamnaceae) and *R. purshiana*	Canada and Italy	**Sexual**: *Asci* octosporous, clavate, up to 24 μm wide; *Ascospores* 20–30(–32) × 6–9 μm, hyaline, hemispores nearly equal or upper longer than lower. **Asexual**: Undetermined	Available	Froidevaux (1972); Barr (2001)
*D. ribesia*	On branches of *Ribes* sp. (Grossulariaceae)	Canada, England, Morocco, New Zealand, Scotland and USA	**Sexual**: *Ascomata* multiloculate, 500–1000 μm diam., rounded pulvinate, the surface plane; upper portion of locules protruding and roughening surface at times; *Walls* composed of vertically oriented rows of cells forming *textura globosa* to *textura prismatica*, blackened externally, often olivaceous to blackish internally, the hyphae penetrating host tissues; locules 60–80 μm diam., 70–100 μm high; *Asci* 60–72 × 11–12 μm; *Ascospores* 15–35 × 4.5–8(–14) μm, hyaline, light dull brown in age, narrowly obovate or elliptic (the ends obtusely pointed), straight o slightly curved, (1–)3–5-septate; no vertical septa formed. **Asexual**: Undetermined	N/A	Barr (1972); MycoBank (2024)
*D. rimincola* ^2^	On dead branches of *Diervilla lonicera* (Caprifoliaceae)	USA	**Sexual**: *Ascomata* 500–1500 μm long or longer by confluence, 38–440 μm wide, 220–270 μm high, elongate or elliptic, erumpent in long rows; *Wall* thick, externally dark brown and 26–39 μm thick, interior layers yellowish or hyaline and 26–35 μm thick, the basal hyaline region forming a raised cushion 52–78 μm deep in mid portion of locule; apex plane or slightly depressed from sides, surface pulverulent dull blackish. *Asci* 50–70 × 12–15 μm, oblong clavate; *Ascospores* inequilateral, 17–25 × 4–5 μm, hyaline, obovate, tapered to an obtuse or pointed base, the upper portion broader than the lower, (1–3–)5–7-septate, constricted at supramedian primary septum, with vertical septum in one or more mid cells. **Asexual**: Undetermined	N/A	Barr (1972)
*D. rufa* ^3^	N/A	N/A	**Sexual**: *Apothecia* on barked wood, widely bleached gregariously sessile, orbicular, then oblong, obtuse, with a dark purple disc, surrounded by a very thin black line, not distinctly marginated, usually convex, roughened, 0.3–2 mm long or wide; *Asci* oval, thickly coated, 60–70 × 30 μm, 8-spored, covered in a gelatinous, yellowish-brown hymen; *Ascospores* ellipsoidal, transversely 5-, longitudinally 1-septate, initially hyaline, then gray-brown, 25–27 × 9–10 μm **Asexual**: Undetermined	N/A	Rehm (1912)
*D. salicis* ^4^	N/A	N/A	N/A	N/A	N/A
*D. sambucina* ^2^	*Sambucus* sp. (Adoxaceae)	USA	**Sexual**: *Ascomata* 230–245 μm diam., 180–200 μm high, often erumpent in long rows; wall 26–33(–50) μm thick; apex short and broadly papillate; *Asci* 52–78 × 15 μm, oblong; *Ascospores* 22.5–27 × 6–7.5 μm, hyaline, obovate (the upper portion broader than the lower), tapered to an obtusely pointed base, often inequilateral, (1–)3–6-septate, constricted at supramedian primary septum, with vertical septum in one or several of mid cells. **Asexual**: Undetermined	N/A	Barr (1972)
*D. schizospora*	On dead stems of *Symphoricarpos orbiculatus* (Caprifoliaceae)	USA (Missouri)	**Sexual**: *Ascomata* solitary or gregarious, innately erupting, orbicular or oblong, flattened, 0.2–1.5 × 0.2–0.7 mm., with parenchymatic context, black, stomatal, rimmed or irregularly dehiscing, dry underclosed, disc dirty white; *Asci* bitunicate, aparaphysate, subcylindrical or clavate, short pedicellate, octosporous, 70–119 × 11–15 μm; *Ascospores* hyaline, fusiform, 1–9-septate, phragmospore or submuriform, gently constricted, deeply constricted to the middle of the septum and easily sessile, 20–44 × 4–6 μm, dichotomous or trichotomous. **Asexual**: Ascomate-like with conidiferous stromata; *Conidia* (blastospores) ovoid or oblong, hyaline, continuous, 4–9 × 2–4 μm, emerging in pockets made of ovoid or pyriform cells 4.6–6 μm diam.	Available	Luttrell (1960)
*D. slippii* ^1^	On dead branches *Pinus albicaulis* (Pinaceae)	USA (Idaho)	**Sexual**: *Ascomata* 250–300 μm diam., pulvinate, often in rows along a branch; apex plane, opening irregularly; *Wall* up to 60 μm thick; *Asci* 48–60 × 11–13 μm, oblong to clavate, parallel from a basal cushion of hyaline cells; *Ascospores* 17.5–24 × 3.5–4.5 μm, hyaline, narrowly obovate, tapered to pointed ends, straight or slightly curved, 3-septate, slightly constricted at primary septum. **Asexual**: Undetermined	N/A	Barr (1972)
*D. sorbi*	On dry branches of *Sorbus aria* (Rosaceae), *S. aucuparia* (Rosaceae), and *Persea americana* (Rosaceae)	France, Germany, Sweden, and Switzerland	**Sexual**: *Asci* elongated, 8-spored, 88 μm long, 24 μm thick.; *Ascsopores* distichous, oblong, constricted in the middle, thicker in the upper part, 6–7-septate, hyaline, 26 μm long, 5 μm thick, **Asexual**: *Perithecia* with spurious, scattered subcaespitosely, under the yellowish epidermis, nestling, globose, striate, of medium size, obtuse to a conical face, prominulum, scarious, tapering to the perithecia; *Styspores* narrowly fusiform, curved, continuous, 14–18 μm long, 2–3 μm thick., guttulates, hyaline	Available	Fuckel (1870); Saccardo (1889)
*D. spartii*	On dead aerial branch of *Spartium junceum* (Fabaceae)	Italy	**Sexual**: *Ascostromata* 370–715 μm high, 320– 340 μm diameter, immersed in the epidermis, solitary or clustered, globose, brown to black, with single locules; *Peridium* 30–50 μm wide; *Asci* 140–175 × 15–22 μm, 8-spored, bitunicate, fissitunicate, cylindro-clavate, short pedicellate, apically rounded, with a small ocular chamber; *Ascospores* 17–21 × 5–7 μm, bi-seriate to multi-seriate, hyaline, aseptate, fusoid to ovoid, smooth-walled, with granular contents, lacking a mucilaginous sheath. **Asexual**: Undetermined	Available	Hyde et al. (2017)
*D. sphaerioides* ^2^	On dry bark, especially on the scars left behind by fallen branches of *Populus tremula* (Salicaceae) and *Populus* spp.	Canada and Germany	**Sexual**: *Asci* oblong, sessile, 8-spored, 100 μm long, 12 μm thick; *Ascospores* distichous, oblong-clavate, constricted in the middle, 5-septate, muriform, hyaline, 19 μm long, 6 μm thick. (in the wider part); *Macrostylospores* with mixed asci, oblong, obtuse on both sides, shortly pedicellate, 5-septate, muriform, pale yellow, 28–36 μm long, 14 μm thick. **Asexual**: *Perithecia* spurious, superficial, gregarious, minute, total 240 μm long, base 128 μm thick, cylindrical-conical, 60 μm wide, 160 μm long, sometimes with a severed beak, fearful; *Stylospores* cylindrical, curved, obtuse on both sides, simple, 6 μm long, 1.5 μm thick.	N/A	Fuckel (1870); Barr (1972)
*D. staphyleae*	On dry branches of *Staphylea pinnata* (Staphyleaceae)	Germany	**Sexual**: *Ascomata* with seriate, often surrounding the entire branch densely and tightly, with a globose-closed subepidermis, then a longitudinal crack v. sometimes breaking out triangularly, black, finally breaking off and falling apart; *Asci* oblong-clavate, octosporous, 100 μm or longer, 12–16 μm wide; Asci distichous, oblong, attenuated on both sides, but obtuse, 4–6-septate, constricted in the middle and unequally bifurcated, with a few muriform-divided pockets, 25–30 × 6–8 μm, hyaline. **Asexual**: Undetermined	N/A	Saccardo and Sydow (1902)
*D. staphylina*	*Staphylea trifolia* (Staphyleaceae)	USA	**Sexual**: *Ascomata* 208–440 μm diam, 117–220 μm high, uni- or multi-loculate; Wall 26 μm thick at sides, up to 90 ju at lower sides at times; apex plane, the pore area pallid under dissecting microscope; *Asci* 65–90 × 12–16 μm, oblong; *Ascospores* 18–22.5 × (4–)5–6(-7) μm, hyaline, obovate (the upper portion broader than the lower), straight to inequilateral, tapered to an obtuse base, (1–)3–5(–7)-septate, slightly constricted at the primary septum, with vertical septum in one or two of mid cells. **Asexual**: Undetermined	N/A	Barr (1972)
*D. stictoides* ^2^	On branches of *Liriodendron* sp. (Magnoliaceae)	USA	**Sexual**: *Ascomata* scattered, connected by thick, black mycelia under host epidermis, immersed, subcuticular, becoming erumpent, pulvinate, uni- to multi-loculate, flat to broadly rounded at apex, subiculum at base, 200–225 µm high, 370–400 µm wide; *Pseudoparaphyses* indistinguishable, sometimes of elongated, compressed pseudoparenchymatic cells between asci, sometimes with thickened septa at both ends, within a gelatinous matrix; *Asci* 110–140 × 17–26 µm, fissitunicate, numerous, arising from hyaline cells or low, disk-shaped hyaline cells, of various numbers in each locule, at irregular heights in hymenial layer, oblong with rounded apex, thin walled throughout or somewhat thick at apex, with eight irregularly biseriate ascospores, short stalked, surrounded by layer of thin gelatinous material, lacking an apical chamber; *Ascospores* 24–34 × 10–13 µm, hyaline to olivaceous, obovate, straight to slightly curved, rounded at apex or sometimes acute, gradually tapered to base, with five to seven transverse septa, septa sometimes slanted, none to six longitudinal septa, sometimes more than one longitudinal septum in a cell, primary septum supramedian, constricted at primary septum, smooth walled with thin gelatinous sheath. **Asexual**: Undetermined	N/A	Ahn and Shearer (1998)
*D. symploci*	On leaves of *Symplocos spicata* (Symplococeae)	Sri Lanka	N/A	N/A	Petrak (1937)
*D. syringae*	On dead branch of *Syringa vulgaris* (Oleaceae)	Russia	**Sexual**: *Stromata* crowded, arranged serially, sometimes subconfluent, bursting through the bark, flattened, various shapes, such as angular-rounded or angular-elongate, smooth, naked, black, very hard when dry, up to 1 mm wide; *Spermatia* ovoid or oval, simple, hyaline, 4–6 μm long, 3 μm thick. **Asexual**: Undetermined	N/A	Karsten (1884)
*D. tamaricis*	On dead leaves and dry branches of *Tamarix gallica* (Tamaricaceae)	Portugal	**Sexual**: *Ascomates* nestling and erupting in the bark, subglobose, 400 μm diam., black; *Asci* ovate or clavate, apex rounded, thickly coated, 90–110 × 40–55 μm, non-coerulescent iodine, octospores; *Ascospores* distichous or irregular, oblong-fusoid, 3-septate, constricted at the middle septum, straight, covered with a mucous layer, made of hyaline olivaceous-brown, 30–42 × 12–14 μm. **Asexual**: Undetermined	N/A	Dennis et al. (1977); Index Fungorum (2024)
*D. taxicola*	On leaves of *Taxus* spp. (Taxaceae)	Canada, England and USA	**Sexual**: *Ascomata* 130–240 μm diam., 145–165 μm high, globose or depressed, immersed with rounded erumpent apex, epiphyllous, thickly scattered; *Wall* 14–30 μm thick, consisting of several layers of polygonal cells, blackened externally; *Apical pore region* stuffed with lighter brown or hyaline cells before maturity; *Asci* 60–96 × 9–14.5 μm clavate, arising from a low dome-shaped cushion of hyaline cells; *Ascospores* 13–18.5 × 3–5 μm, hyaline or yellowish, narrowly elliptic or obovate, tapered to pointed ends, straight to slightly curved, (l–)3-septate, not constricted at septa; contents minutely guttulate, smooth-walled. **Asexual**: *Pycnidia* immersed, depressed, 418–435 μm diam., 243–352 μm high, multiloculate; *Conidiophores* short, 7–15 × 1.5–2 μm; *Conidia* 3–5 × 1 μm, hyaline, one-celled.	N/A	Barr (1972)
*D. thujae*	On cone scales of *Thuja occidentalis* (Cupressaceae)	Great Britain and USA	**Sexual**: *Ascomata* 130–245 μm diam., nearly globose, single or few grouped together and connected by hyphae; wall 20–50 μm thick, thickest at base; apex rounded papillate; *Asci* 37.5–63 × 18–27 μm, broadly oblong; *Ascospores* 20–30 × 6–9 μm, yellowish brown, obovate, with ends obtuse, straight to inequilateral, (3–)5 (–7)-septate, constricted at the primary and less at the secondary septa, with vertical septum (rarely two) in mid cells, occasionally extending into the apical cell; thin gelatinous coating 1.5–2 μm thick at times surrounding ascospores. **Asexual**: Undetermined	N/A	Barr (1972)
*D. vaccinii*	On dry stems and branches of *Vaccinium uliginosum* (Ericaceae)	Switzerland	**Sexual**: *Ascomata* breaking out, finally very prominent, gregarious, usually covering the entire branches, brownish-black, 1 mm diam., rounded but usually elliptical and irregular, with a raised edge, swollen, concave apex, first carbonaceous, concolorous, finally irregularly fissured and exposed to a dirty disc; *Asci* clavate, 8-spored, 74 μm long, 12 μm thick (in the clavula); *Ascospores* filled, oblong-ovate, obtuse on both sides, slightly curved, continuous, hyaline, 12 μm long, 6 μm thick. **Asexual**: Undetermined	N/A	Fuckel (1875)
*D. valdiviana*	On branches of *Saxegothaea conspicua* (Podocarpaceae)	Chili (Biobío)	**Sexual**: *Ascomata* solitary or gregarious, intraepidermal, innately erupting, flattened, black, irregularly orbicular, 100-300 μm diam.; *Asci* bitunicate, broadly clavate or obovoid, 8-spored, 30–40 × 16–20 μm, aparaphysate, arranged in pale multiseriate pockets; *Ascospores* distichous or tristichus, ellipsoid or clavate, hyaline, phragmospore or muriform, gently constricted at the middle of the septum, 12–16 × 6–7 μm. **Asexual**: Undetermined	N/A	Butin (1973)
*D. versiformis* ^2^	On dead branch of *Sorbus sitchensis* (Rosaceae)	British Columbia	**Sexual**: *Ascomata* 440–660μm diam., 330–440 μm high, elliptic or rounded from above, single locule; *Wall* 60–80 μm thick at sides and upper surface, up to 180 μm deep at base; *Asci* polysporous, 120–140 × 20–26 μm, broadly oblong, pararella; *Ascospores* 8–10 × 3.5–4.5 μm, 1-celled or 1-septate, 17–25 × 6–9 μm, 3–5-septate, hyaline to brown, elliptic or obovate, often broadest above and tapered to the pointed base, constricted at primary septum or often all septa, with vertical septum in one or more mid cells. **Asexual**: Undetermined	N/A	Barr (1972); Barr, (2001)
*D. viburnicola*	On dead leaf of *Viburnum tinus* (Adoxaceae)	Italy	**Sexual**: Undetermined **Asexual**: *Conidiomata* pycnidial, globose with long neck, brown, to 250 μm diam, with central ostiole, exuding a creamy conidial mass; *Conidiophores* reduced to conidiogenous cells lining the inner cavity, hyaline, smooth, ampulliform to doliiform, 5–7 × 5–6 μm, with central phialidic locus; *Conidia* hyaline, smooth, guttulate, subcylindrical, apex obtuse, tapering to a truncate hilum (6.5–)8–10(–13) × (2–)2.5(–3) μm	Available	Crous and Groenewald (2016)
*D. viticola*	Fruit (grapes) of *Vitis vinifera* cv. Malvasia (Vitaceae)	Spain	**Sexual**: Undetermined **Asexual**: *Mycelium* composed of hyaline, branched, strongly septate hyphae, smooth- and thin-walled, swollen at septa, 3.5–6 μm diam, becoming monilliform and dark brown with age due to the production of solitary to catenate chlamydospores of up to 20 μm diam, with some segments remaining hyaline or nearly so; *Conidiogenous cells* integrated on hyphae, intercalary or terminal, inconspicuously to conspicuously denticulate; *Conidia* holoblastic, at first synchronously produced in small groups on lateral protrusions of the hyphae on short (1 μm long) conic-truncate denticles, and later percurrently produced along the hyphae and on side branches from larger denticles (1.0–1.5 μm long); *Conidia* hyaline at first, mostly aseptate, sometimes septate at the middle and slightly constricted at septa, smooth and thin-walled, variable in shape but mostly ellipsoidal, clavate at both ends when septate, 5–17 × 4–10 μm, becoming dark brown and thick-walled with the age, smooth-walled to granulose due to deposition of a dark pigment on the cell surface, 12–20 × 4–12 μm. *Microcyclic conidia* produced by budding of both hyaline and pigmented conidia, produced singly or in chains of up to 4, at one or both ends but sometimes laterally, being smaller than the primary ones. Endoconidia also present in hyaline segments of hyphae, ellipsoidal, hyaline, 4–6 × 3–4 μm.	Available	Crous et al. (2015); Crous et al., (2022)
*D. wolfii* ^1^	On branches of *Oxydendmm arboretum* (Ericaceae)	USA (North Carolina)	**Sexual**: *Ascomata* 200–250 μm diam, 150–200 μm high, depressed globose, grouped at times in dark reddish-brown areas on a branch, or scattered; *Wall* 20–55 μm thick; *Asci* 55–70 × 12–14.5 μm, oblong, parallel from a flattened basal cushion of hyaline cells; *Ascospores* 17.5–22 × 4.5–6 μm, hyaline, narrowly obovate, tapered to obtuse ends, inequilateral to slightly curved, (2–)3–5-septate, constricted at primary septum. **Asexual**: Undetermined	N/A	Barr (1972)
*D. xylostei*	On dry trunks and thinnest branches of *Lonicera xylosteum* (Caprifoliaceae)	Germany	**Sexual**: *Spermogonia* like a fungus, but smaller; Spermatia oblong-ovate, continuous, hyaline, 6–8 μm long, 3–4 μm thick. The cups of the ascigerus are sparse, bursting through the cracks in the bark, ½ μm long., circular or oblong, flat, sometimes with a protruding or irregular edge, black, dirty inside, cornified; *Asci* oblong, subsessile, unequally bisected, 4-septate, constricted below the middle, a slightly thicker part uniseptate, a narrower part biseptate, straight, hyaline, 20 μm long., 4–5 μm thick. **Asexual**: Undetermined	N/A	Fuckel (1871)

N/A, no information available

^1^Based on the Species Fungorum; the taxon was synonymized under other genera.

^2^Based on the MycoBank database; the taxon was synonymized under other genera.

^3^The species shows as Nom. inval., Art. 36.1(c) (Melbourne) in Index Fungorum, but this became legitimate in MycoBank.

^4^The species is regarded as an illegitimate name according to MycoBank (2024), but it was shown as valid in Index Fungorum (2024).

Although the sexual morph of *Dothiora* species has been delimited according to morphological criteria by having one or more septate or muriform ascospores (Thambugala et al., 2014; Crous and Groenewald, 2017), many species with aseptate ascospores were also classified as *Dothiora* based on morphological characteristics and phylogenetic analyses (Hyde et al., 2016; Hyde et al., 2017; Boonmee et al., 2021). Most *Dothiora* species with hyaline to pale brown, one or more septate or muriform ascospores, form a separate clade without statistical support (**Figure 1**), while three new taxa, *D. capparis*, *D. rhapontici*, and *D. uzbekistanica*, cluster with *D. buxi*, *D. coronillae*, *D. coronillicola*, and *D. spartii*, which also have hyaline, aseptate, and fusoid to ovoid ascospores with 89% ML and 0.99 PP statistical support in different clades, agreeing with previous studies (Boonmee et al., 2021). *Dothiora rhapontici* and *D. buxi* differ from related species in having polysporous asci, while other species have octosporous asci. These species can be distinguished from each other based on their asci and ascospores sizes; moreover, *D. capparis* can be distinguished from *D. coronillae*, *D. coronillicola*, *D. spartii*, and *D. uzbekistanica* by the presence of a mucilaginous sheath surrounding the ascospores. *Dothiora cactacearum* also clustered among these species; however, its morphological characteristics cannot be compared with others, as it is known only by its asexual morph. *Dothiora buxi* is phylogenetically close to *D. cactacearum*, and both species have similar asexual morphs to the generic, as described by Thambugala et al. (2014) and Crous and Groenewald (2017). The morphology of ascospores of *Dothiora buxi*, *D. capparis*, *D. coronillae*, *D. coronillicola*, *D. rhapontici*, *D. spartii*, and *D. uzbekistanica*, was clearly distinct from that of most *Dothiora*; however, only this evidence did not have enough support to accommodate a distinct lineage in Dothideaceae. Furthermore, an asexual morph of these species has not been cultured or reported to verify its morphological features, except for *D. buxi*. Due to the fact that most of the available sequences of *Dothiora* are only LSU and/or ITS, their taxonomic position remains uncertain. The taxonomic classification of *Dothiora* species is still incomplete; further investigations of freshly collected specimens in different regions and sequence data are needed to better understand their natural classification.

Crous et al. (2018b) transferred *Kabatina mahoniae* A.W. Ramaley to *Dothiora* as *D. mahoniae*. According to the multigene analyses herein (**Figure 1**), *Dothiora mahoniae* (strain CBS 264.92) clustered with *Neodothiora populina* Crous, G.C. Adams & Winton (strain CBS 147087) with no statistical support for this relationship and separated from *Dothiora* species, which is consistent with Crous et al. (2020). Based on a comparison of morphology, *Dothiora mahoniae* fits well with the generic concepts of *Kabatina* rather than *Dothiora* and *Neodothiora* (Ramaley, 1992; Thambugala et al., 2014; Crous and Groenewald, 2017; Crous et al., 2020). In addition, only LSU and ITS are available for *D. mahoniae* in GenBank. Thus, the species is retained until more evidence of fresh collections with DNA sequence data is available to resolve its phylogenetic placement within the family.

In this study, three new species of hyaline-spored *Dothiora* (*D. capparis*, *D. rhapontici*, and *D. uzbekistanica*) are described and illustrated. It is interesting to note that *Dothiora* have morphological variability in their spores. Thus, it is inadequate to determine *Dothiora* spp. based solely on morphological data. It can be seen that phylogenetic analyses are necessary to confirm morphology-based identifications and detect species new to science. Many *Dothiora* on the list (**Table 2**) have not been verified yet based on the molecular data; however, these species have characteristics that match the generic description. Additionally, the classification of several species remains unclear due to the variability in some morphological characters, a lack of molecular information regarding protein-coding genes, and no sexual–asexual links. Hence, these species are not excluded from *Dothiora* until increased taxon samplings and sequence data are available. Further sampling is necessary to improve our knowledge of the diversity, ecology, and impacts of hyaline-spored *Dothiora* species on flowering plants in arid and semi-arid habitats.

## Data availability statement

The datasets presented in this study can be found in online repositories. The names of the repository/repositories and accession number(s) can be found below: https://www.ncbi.nlm.nih.gov/genbank/, ITS: PP086677, PP086678, PP086679, PP086680, PP086681, PP086682, PP086683 and PP086684; LSU: PP086685, PP086686, PP086687, PP086688, PP086689, PP086690 and PP086691; SSU: PP086692, PP086693, PP086694, PP086695, PP086696 and PP086697; TEF1: PP084936, PP084937, PP084938, PP084939, PP084940, PP084941, PP093832 and PP093833.

## Author contributions

CS: Data curation, Formal analysis, Investigation, Methodology, Software, Validation, Visualization, Writing – original draft, Writing – review & editing. SH: Conceptualization, Formal analysis, Project administration, Supervision, Visualization, Writing – review & editing. SK: Formal analysis, Investigation, Visualization, Writing – review & editing. JK: Data curation, Investigation, Software, Supervision, Visualization, Writing – original draft, Writing – review & editing. MY: Investigation, Resources, Visualization, Writing – review & editing. YG: Conceptualization, Data curation, Investigation, Project administration, Resources, Supervision, Visualization, Writing – review & editing. AA: Investigation, Resources, Visualization, Writing – review & editing. NS: Conceptualization, Data curation, Formal analysis, Funding acquisition, Investigation, Methodology, Project administration, Supervision, Validation, Visualization, Writing – original draft, Writing – review & editing.
